# Rigid Biobased Vinylogous Urethane Vitrimers from d‑isosorbide/Furfural-Derived Monomers

**DOI:** 10.1021/acspolymersau.6c00063

**Published:** 2026-06-19

**Authors:** Ondrej Kopilec, Jiri Hodan, Ondrej Sedlacek, Hynek Beneš

**Affiliations:** † Department of Physical and Macromolecular Chemistry, Faculty of Science, 112302 Charles University, 128 00 Prague 2, Czech Republic; ‡ Institute of Macromolecular Chemistry of the Czech Academy of Sciences, 162 00 Prague 6, Czech Republic

**Keywords:** vitrimers, covalent adaptable networks, d-isosorbide, biobased polymers, circular thermosets

## Abstract

Biobased covalent adaptable networks (CANs, vitrimers) that combine high glass transition temperatures, mechanical robustness, and reprocessability remain scarce, particularly in vinylogous urethane vitrimer chemistry. Herein, we report the synthesis of rigid vinylogous urethane CANs derived from biobased d-isosorbide bis-acetoacetate and the furfural-based diamine propane-2,2-di­(2-furylmethylamine) (PDFA). The materials were prepared by a solvent-free bulk curing strategy using mixtures of di- and trifunctional amines, enabling direct formation of cross-linked networks without additional processing solvents. The use of PDFA significantly increased the renewable carbon content while maintaining the rigidity required for glassy high-performance materials. The resulting networks exhibited tunable thermomechanical properties depending on amine structure and composition, with glass transition temperatures up to 125 °C and thermal stability up to 290 °C. Stress-relaxation experiments confirmed fast catalyst-free bond exchange in the rubbery state, with relaxation occurring on the scale of tens to hundreds of seconds and activation energies dependent on the diamine structure. Selected materials displayed efficient reprocessability by compression molding while largely preserving their thermal and tensile properties after recycling. These results demonstrate that combining d-isosorbide with a rigid furfural-derived diamine is an effective strategy toward biobased, glassy vinylogous urethane vitrimers that unite high renewable carbon content, elevated service temperature, and recyclability.

## Graphical abstract

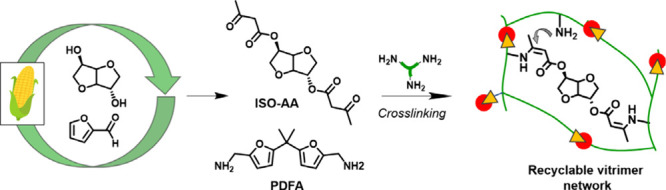

## Introduction

Vitrimers represent an emerging class of cross-linked polymer materials that bridge the gap between traditional thermosets and thermoplastics due to the presence of dynamic covalent bonds between polymer chains.^1,2^ While traditional thermosets are valued for their excellent mechanical strength, temperature, and chemical resistance, their permanently cross-linked network renders them physically nonrecyclable. Conversely, thermoplastics can be reprocessed at elevated temperatures but often lack the mechanical strength required in high-performance applications. Vitrimers resolve this dichotomy, as they behave as thermoset-like permanent polymer networks at service temperatures, while being reprocessable upon applying external or internal stimuli, such as heat.^3^ At elevated temperatures, vitrimer materials undergo dynamic bond exchange reactions, such as transaminations or transesterifications, enabling the rearrangement of the polymer network while maintaining overall cross-link density.

Compared to other classes of covalent adaptable networks (CANs), e.g., dissociative Diels–Alder networks, where the cross-link density drops significantly during thermal rearrangement, vitrimers typically follow an associative mechanism, where the bond exchange occurs simultaneously, preserving the network integrity and ensuring that the material retains its structural properties throughout reprocessing.^4^ Several types of dynamic covalent bond exchange reactions have been studied in vitrimers,^2^ such as transesterifications,^5^ transaminations,^6^ imine or disulfide exchange reactions,^7,8^ and transalkylations.^9^ In particular, transamination of vinylogous urethane networks represents a widely studied chemistry in vitrimers,^6,10^ due to its straightforward implementation, catalyst-free nature, and low topology freezing temperature (*T*_v_, vitrification temperature), which is the temperature at which the dynamic exchange reactions become fast enough for the polymer network to flow like a viscoelastic liquid.

Despite their promising potential for a circular economy, the majority of vitrimers reported to date rely on monomers derived from fossil-based feedstocks.^11^ As the industry moves toward sustainable manufacturing, it is critical to develop novel vitrimer materials that utilize renewable carbon resources. As a result, several biobased vitrimers systems have been reported,^11–13^ derived from renewable feedstocks such as vegetable oils (e.g., epoxidized soybean oil),^14^ terpenes (e.g., pinene, limonene),^15^ lignin derivatives (e.g., vanillin, eugenol),^16,17^ or saccharide derivatives (e.g., starch, cellulose, and 2,5-furandicarboxylic acid).^18,19^

In recent decades, starch-based d-isosorbide has emerged as a prominent biobased building block that aligns with the core principles of green chemistry due to its sustainability, nontoxicity, and biodegradability.^20^ d-Isosorbide is a rigid bicyclic diol that can be modified into a variety of sustainable polymer materials. Its rigidity helps to maintain a high glass transition temperature of isosorbide-based materials.^21^ Despite its potential, there are only few reports on the use of d-isosorbide as building blocks in vitrimer materials. As an example, Ma et al. reported on a biobased epoxy vitrimer synthesized from isosorbide-derived epoxy resins and aromatic diamines containing disulfide bonds, demonstrating fast stress relaxation and controllable shape memory.^22^ Adjaoud et al. demonstrated the synthesis of fully biobased polybenzoxazine vitrimers from isosorbide that exhibit high glass transition temperatures and excellent degradability through consecutive esterification and Mannich-like reactions.^23^ Liguori et al. explored the digital light processing 3D printing of isosorbide- and vanillin-based thermosets, highlighting how the combination of ester and imine functionalities influences both the printability and the chemical recyclability of the resins.^24^ Furthermore, Li et al. developed recyclable high-performance thermosetting plastics based on isosorbide and Diels–Alder chemistry, further demonstrating the suitability of rigid isosorbide-derived structures for recyclable thermoset applications.^25^ Matt et al. prepared covalent adaptable polymethacrylate networks containing isosorbide levulinate side groups cross-linked through dynamic acylhydrazone bonds, achieving enhanced glass transition temperatures, thermal stability, and chemical recyclability.^26^ Furthermore, several recent papers report on the synthesis of d-isosorbide-based vinylogous urethane vitrimers.^27,28^ As an example, Yang et al. developed hybrid d-isosorbide networks that integrate both epoxy chemistry with vinylogous urethane cross-linking.^27^ However, these systems still rely on petroleum-derived amine cross-linkers like triethylenetetramine (TETA) or Jeffamine T-403, which limits their overall sustainability. Recent efforts by Fanjul-Mosteirín and Odelius addressed the low biobased carbon content by incorporating renewable polyamines (Priamines) to increase biocontent,^29^ yet the flexible, long-chain aliphatic nature of these fatty-acid-derived amines resulted in materials with low glass transition temperatures (*T*_g_), thereby compromising the service temperature window for structural use.

In this work, we have designed and synthesized novel biobased glassy vinylogous urethane vitrimers with high *T*_g_, which pair d-isosorbide monomer with the rigid, furfural-derived diamine PDFA (propane-2,2-di­(2-furylmethylamine)). This combination allows for a purely vinylogous urethane network that achieves both maximum renewable carbon content and high mechanical rigidity required for demanding applications, all while maintaining efficient, catalyst-free recyclability. The vitrimers have been studied by a plethora of analytical techniques, including DSC, DMTA, and tensile testing, exploring the effect of comonomer structure and ratio on thermomechanical properties, aiming for biobased rigid high-*T*_g_ vitrimers. Our results demonstrate that these materials possess the mechanical toughness and chemical resistance typical of traditional thermosets, yet they remain reprocessable at higher temperatures due to the low activation energy of their transamination reactions.

## Experimental Section

### Materials

All reagents and solvents were used as received without further purification. d-Isosorbide (98%), *tert*-butyl acetoacetate (≥98%), and benzylamine (99%) were purchased from Merck. Furfurylamine (>98%), *m*-xylylenediamine (XYDI, > 99%), tris­(2-aminoethyl)­amine (TREN, 96%), and isopropanol (>99.5%) were purchased from TCI Europe. Trimethylolpropane tris­[poly­(propylene glycol), amine-terminated] ether (Jeffamine T-403, JEFF) was kindly provided by Hunstman. Acetone (pure), dichloromethane (pure), HCl (conc. 37%), tetrahydrofuran (pa), and NaOH were purchased from Lach-Ner.

### Synthesis of Isosorbide-bis-acetoacetate (ISO-AA)

In a 250 mL round-bottom flask, d-isosorbide (40 g, 0.27 mol) and *tert*-butyl acetoacetate (140 g, 0.89 mol) were mixed and then heated at 130 °C for 24 h. The side product (*tert*-butanol) was continually distilled off from the reaction mixture, first at atmospheric pressure and later at reduced pressure (450 mbar). The progress of the reaction was monitored by TLC on silica (5% MeOH in dichloromethane). Once the reaction was completed, the unreacted *tert*-butyl acetoacetate was removed by rotary evaporation at 40 °C under reduced pressure. A pale-yellow oil (ISO-AA, 83 g, 97% yield) with a tendency to crystallize at room temperature was obtained. The characterization data are consistent with those reported in the literature.^29^ ^1^H NMR (CDCl_3_, TMS) δ, ppm: 12.23 (s, minor enol tautomer), 11.85 (dd, minor enol tautomer), 5.26–5.21 (m, 1H), 5.19 (dd, 1H), 4.82 (t, 1H), 4.54 (dd, 1H), 4.01–3.92 (m, 3H), 3.85–3.80 (m, 1H), 3.51–3.48 (s, 4H), 2.29–2.25 (s, 6H). HRMS (ESI): calculated for [M+Na^+^] *m*/*z* = 337.0894, measured *m*/*z* = 337.0893.

### Synthesis of Propane-2,2-di­(2-furylmethylamine) (PDFA)

PDFA was prepared using a modified procedure of Bu et al.^30^ Concentrated HCl (250 mL) in a 1 L flask was cooled to subzero temperature in an ice-salt bath. Subsequently, furfuryl amine (116.5 g, 1.2 mol) was added dropwise while stirring. After the addition, the mixture was allowed to spontaneously warm up to room temperature, followed by the addition of acetone (29 g, 0.5 mol). The reaction mixture was left stirring overnight at room temperature, with a color change observed. Finally, the formed white precipitate was collected and washed several times with isopropanol. The precipitate was dried in a vacuum oven overnight at 60 °C, then dissolved in NaOH (500 mL, 3M) and extracted with ethyl acetate (3 × 200 mL). The organic layers were combined, washed with brine, residual water was removed with MgSO_4_, and the solvent was evaporated using rotational evaporation. 66 g (57% yield) of PDFA product was obtained in the form of a brown oily liquid. The characterization data are consistent with those reported in the literature.^30^ ^1^H NMR (DMSO-*d*_6_, TMS) δ, ppm: 6.06–5.97 (d, 4H), 3.60 (s, 4H), 1.54 (s, 6H). ^13^C NMR (DMSO-*d*_6_, TMS) δ, ppm: 158.40, 156.58, 105.61, 105.02, 39.37, 37.16, 26.74. HRMS (ESI): calculated for [M+Na^+^] *m*/*z* = 257.1255, measured *m*/*z* = 257.1250.

### Preparation of CANs

The networks were prepared by various combinations of diamine (XYDI or PDFA) and triamine (TREN or JEFF) with ISO-AA, always with a 10% excess of NH_2_ groups to ketones. The diamine/triamine molar ratios of 1/1 and 3/1 were tested. First, the amines were mixed together in a polypropylene cup. Then, ISO-AA was quickly added, and all components were homogenized at room temperature for 20 s at 2 500 rpm using a mechanical stirrer (Witeg Labortechnik HS-120A). Then the reactive mixture was placed into the oven, cured at 90 °C for 24 h, and subsequently postcured at 130 °C for 30 min. In this way, 8 different vinylogous urethane CANs were prepared (Table 1). The cured samples were ground to fine powder using a mill (IKA A 10 basic), and then hot-pressed with a force of 50 kN to square- and dog-bone-shaped molds. The hot-pressing conditions (i) P1T and P3T samples, 140 °C for 20 min; (ii) X1T and X3T samples, 150 °C for 30 min; (iii) P1J and P3J samples, 120 °C for 20 min; and (iv) X1J and X3J samples, 170 °C for 15 min

**Table 1:** Compositions of Monomer Feeds for Prepared CANs

·sample ID	diamine	triamine	di-/tri-amine^a^	iso-AA/diamine/triamine (wt %)	iso-AA/diamine/triamine^a^	Bio C (%)
P1T	PDFA	TREN	1/1	67/19/14	1/0.44/0.44	45.5
P3T	PDFA	TREN	3/1	63/30/7	1/0.73/0.25	52.2
X1T	XYDI	TREN	1/1	72/14/15	1/0.44/0.44	29.8
X3T	XYDI	TREN	3/1	70/22/8	1/0.73/0.25	28.1
P1J	PDFA	JEFF	1/1	53/15/32	1/0.44/0.44	41.4
P3J	PDFA	JEFF	3/1	55/26/19	1/0.73/0.25	49.7
X1J	XYDI	JEFF	1/1	55/11/34	1/0.44/0.44	26.8
X3J	XYDI	JEFF	3/1	60/19/21	1/0.73/0.25	26.6

a Initial molar ratio.

**Reprocessing** of the prepared networks was performed via compression molding using the above-mentioned conditions

The biobased carbon content (% Bio C, Table 1) was calculated as the ratio of biobased carbon to the total carbon in the network. In this calculation, only isosorbide and furfurylamine were considered as biobased components of CANs (Scheme S1).

### Characterizations

*Nuclear magnetic resonance (NMR)* spectra were recorded on a Bruker Avance MSL 400 MHz spectrometer at room temperature. Samples were dissolved in 0.5 mL of CDCl_3_ or DMSO-*d*_6_, respectively.

*HRMS spectrum* of ISO-AA was measured on a Q–TOF Bruker in ESI+ mode. HRMS spectrum of PDFA was measured on LC-QTOF Agilent also in ESI+ mode.

*Infrared (FTIR) spectra* were measured on a Thermo Scientific iN10 spectrometer. The homogenized powdered samples were spread onto the instrument measurement area. The ATR-FTIR spectra were collected using a diamond probe as an average of 256 scans.

*Thermogravimetric analysis (TGA)* was performed on a Pyris 1 TGA thermogravimetric analyzer (PerkinElmer, USA). The samples were heated at a rate of 10 °C/min from 25 to 800 °C under a nitrogen atmosphere.

*Dynamic mechanical and thermal analyses (DMTA)* were conducted on an ARES-G2 rheometer (TA Instruments, USA). The temperature dependence of the complex shear modulus of rectangular samples was measured by oscillatory shear deformation in the temperature range of 25–200 °C at a temperature ramp rate of 3 °C/min with 0.01% oscillation strain and 1 Hz frequency. The main transition temperature (*T*_α_) was evaluated as the maximum of the tan δ peak.

*Differential scanning calorimetry (DSC)* measurements were performed on a DSC calorimeter Q 2000 (TA Instruments). The instrument was calibrated for heat flow and temperature using indium. Samples of approximate weight 10 mg were sealed in aluminum pans and measured in a heat–cool-heat cycle with a 10 °C/min temperature ramp in the temperature range of 0–200 °C. The glass transition temperature (*T*_g_) was determined as the midpoint on the second heating scan curve.

*Stress-relaxation experiments* were conducted on an ARES-G2 rheometer (TA Instruments, USA) on rectangular samples in torsion. An axial force of 0.2 N and a deformation of 4% within the linear viscoelastic (LVE) region were applied, and the time evolution of relaxation modulus, *G*(*t*), was monitored at different isotherms. The *G*(*t*) curves were fitted using (i) Maxwell model (eq 1):

1 $$G\left(t\right)=G_{0}\;\exp\left(-t/\tau^{*}\right)$$

where *G*_0_ is an initial modulus and τ* is a characteristic relaxation time, and (ii) Kohlrausch–Williams–Watts (KWW) model (eq 2):^31^

2 $$G\left(t\right)=G_{0}\;\exp\left[{-\left(t/\tau^{*}\right)}^{\beta }\right]$$

where β is a stretching coefficient (0 < β ≤ 1). The average relaxation time, ⟨τ⟩, is then calculated according to (eq 3):^32^

3 $$\langle \tau \rangle =\frac{\tau^{*}\Gamma \left(1/\beta \right)}{\beta }$$

where Γ is the gamma function.

*Tensile tests* were measured according to the ISO 527 test method at room temperature with a cross-head speed of 2 mm/min with a 1 kN load cell on an Instron model 6025/5800R (Instron Limited, High Wycombe, UK). Dogbone-shaped specimens of 1 mm thickness (the total length of the specimen was 75 mm; the length and width of the narrowed part were 25 and 4 mm, respectively) corresponded to the ISO 527–4/5A type. Values are expressed as the means ± standard deviations of at least six specimens. The significance of the differences in the results was evaluated using one-way analysis of variance (ANOVA, α = 0.05) and Tukey’s HSD test for post hoc analysis.

*Swelling ratio and gel content* of the synthesized CAN were determined from the extraction test. A piece of dried sample was weighed (*m*_dry_), then placed into tetrahydrofuran (THF), allowed to swell at RT until equilibrium was reached equilibrium, and then weighed in the swollen state (*m*_swollen_). After the test, the samples were dried in a vacuum oven until constant weight (*m*_exct_). The mass swelling ratio (*Q*_m_) was determined according to

$$Q_{m}=\left(m_{\mathrm{swollen}}-m_{\mathrm{dry}}\right)/m_{\mathrm{dry}}$$

The gel content (*w*_gel_ in wt %) was determined according to

$$w_{\mathrm{gel}}=100\cdot m_{\mathrm{exct}}/m_{\mathrm{drt}}$$

## Results and Discussion

### Synthesis of Biobased Monomers

The successful preparation of high-*T*_g_ biobased vinylogous urethane vitrimers required two rigid monomers: isosorbide bisacetoacetate (ISO-AA) and the furfural-derived diamine propane-2,2-di­(2-furylmethylamine) (PDFA). As the overall concept of this work combines renewable feedstocks with practical and solvent-minimized processing, particular attention was paid to developing efficient and more sustainable synthetic procedures for both compounds.

Although isosorbide-based bisacetoacetates have already been employed in a limited number of vitrimer reports,^27–29^ their synthesis typically relies on the use of aromatic solvents (e.g., toluene, xylene), which is undesirable from an environmental perspective. Here, in contrast, we synthesized ISO-AA in bulk by transesterification reaction of d-isosorbide with *tert*-butyl acetoacetate (Figure 1), giving the desired monomer in high yield (>97%). It is worth noting that the excess of *tert*-butyl acetoacetate could be easily recovered by distillation and used in further synthesis batches. The structure and purity of ISO-AA was confirmed by ^1^H NMR (Figure 1), FTIR, and ESI-MS (Figures S1–S2) measurements, which verified the substitution of both hydroxyl groups of d-isosorbide.

**Figure 1:**
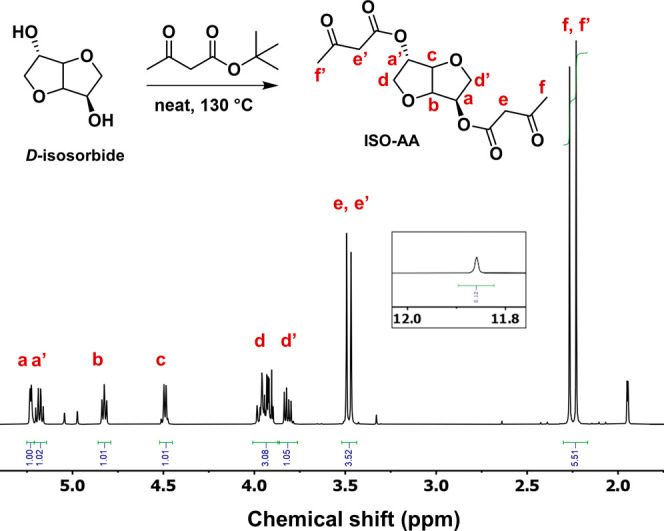
Reaction scheme for the solvent-free synthesis of isosorbide bis­(acetoacetate) (ISO-AA, top). ^1^H NMR spectrum of ISO-AA in CDCl_3_.

In parallel, PDFA was synthesized as a rigid biobased diamine intended to increase both the renewable carbon content and the structural rigidity of the vitrimer networks, reflected in their high *T*_g_ values. To the best of our knowledge, the use of PDFA in vinylogous urethane vitrimers has not been reported yet. In analogy to the isosorbide-based monomer, the reported PDFA synthesis^30^ was optimized toward a more environmentally friendly workup, replacing petroleum-derived chemicals used in extraction with more sustainable ethyl acetate, without compromising the isolated yield. The structure of PDFA was confirmed by ^1^H and ^13^C NMR spectrometry (Figures S3–S4). The combination of ISO-AA and PDFA thus provides a promising monomer platform for the construction of rigid biobased vitrimers with high renewable carbon content and glassy thermomechanical behavior. Furthermore, to compare the herein developed PFDA-based vitrimers with reported nonrenewable glassy vitrimers, commercial m-xylylene diamine (XYDI) was used in control experiments. Finally, commercial trifunctional amines tris­(2-aminoethyl)­amine (TREN) and polymeric triamine Jeffamine T-403 (JEFF) have been used as cross-linking agents for our study.

### Covalent Adaptable Networks (CANs)

Having established efficient routes to both monomeric building blocks, we next investigated their use in the preparation of biobased vinylogous urethane vitrimers. One of the practical goals of the work is to verify whether the isosorbide-based CANs can be considered as a potential replacement for conventional thermosetting resins, typically glassy epoxy materials, which are widely used as cast systems in electronics and composites. For this reason, we selected amine hardeners that can provide highly cross-linked materials (Scheme 1). In accordance with environmental requirements to reduce the carbon footprint of polymers, the prepared CANs were designed with a maximum content of biobased raw materials. From this perspective, herein, the use of aromatic biobased diamine PDFA for the preparation of glassy CANs. Moreover, by using it in combination with ISO-AA, the amount of carbon from biomass (bio C) increased significantly (Table 1). Unfortunately, a suitable biobased alternative to the trifunctional amine TREN has not yet been found, and therefore, 100% biobased CANs have not been achieved.

**Scheme 1:**
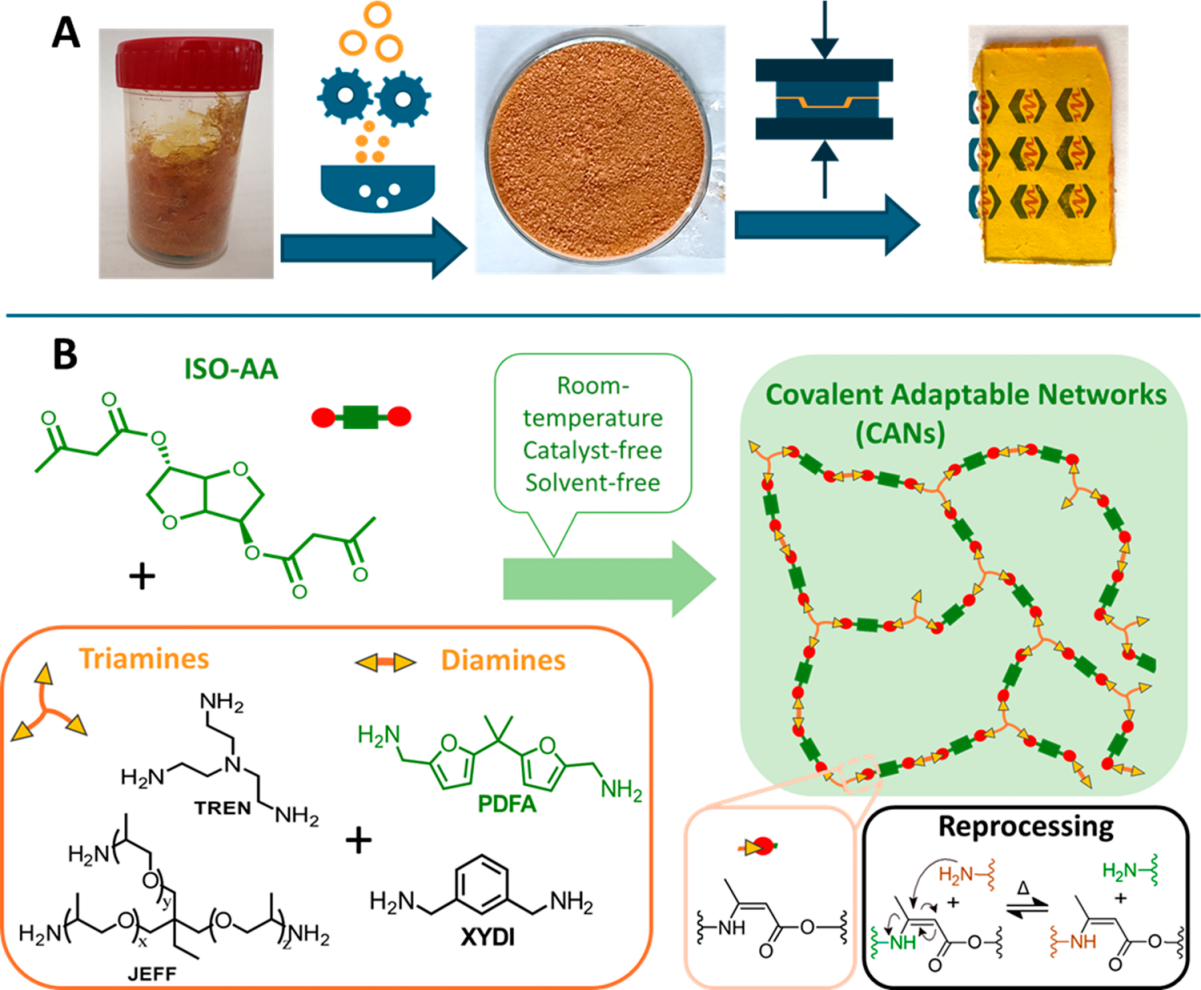
(A) Preparation Route of Covalent Adaptable Networks (CANs) Demonstrated on P1T Sample: (i) Bulk Polymerization Step in Plastic Crucibles, (ii) Grinding to a Fine Powder, and (iii) Its Subsequent Processing by Compression Molding; (B) Reaction Scheme of Isosorbide-bis-acetoacetate (ISO-AA) and Two- And Three-Functional Amines (PDFA, XYDI, Jeffamine T-403–JEFF, and TREN) Forming Glassy CANs (The Fabricated CANs Can Be Reprocessed upon Heating by Amine Exchange of Vinylogous Urethanes)

Furthermore, a combination of di- and trifunctional amines was always chosen to reduce the initial viscosity of the reaction mixture and adjust the appropriate reactivity. This approach allowed us to perform polymerization in bulk and create CAN networks directly without the need for additional solvent removal. In contrast, all previous studies have always carried out the preparation of biobased vinylogous urethane CANs in the presence of a solvent,^29,33^ which limits their scale-up.

Herein, it is shown that a mixture of di- and tri- amines can be homogenized in bulk with ISO-AA, avoiding the use of harmful solvents. Moreover, the reaction in bulk proceeded very rapidly; after curing at 90 °C (24 h) and short (30 min) postcuring at 130 °C, the FTIR spectra (Figure 2) showed complete disappearance of acetoacetate groups, the formation of the characteristic C=C and C=O ester bands of the vinylogous urethane bonds at 1593 cm^–1^ and 1647 cm^–1^, respectively.^28^ The presence of amino groups due to excess of amine functionalities was also confirmed as the N–H vibrations at ca. 3300 cm^–1^. The ground materials (Figure S5) thus exhibited no residual unreacted acetoacetate groups and the presence of free amino groups available for transamination exchange, confirming that the CANs were successfully created according to Scheme 1. The CAN powders were processed via compression molding, producing homogeneous 0.7 mm (Figure S6) or 1.3 mm (for stress relaxation test) thick plates. This solvent-free approach has demonstrated that the developed materials have potential applicability on an industrial scale.

**Figure 2:**
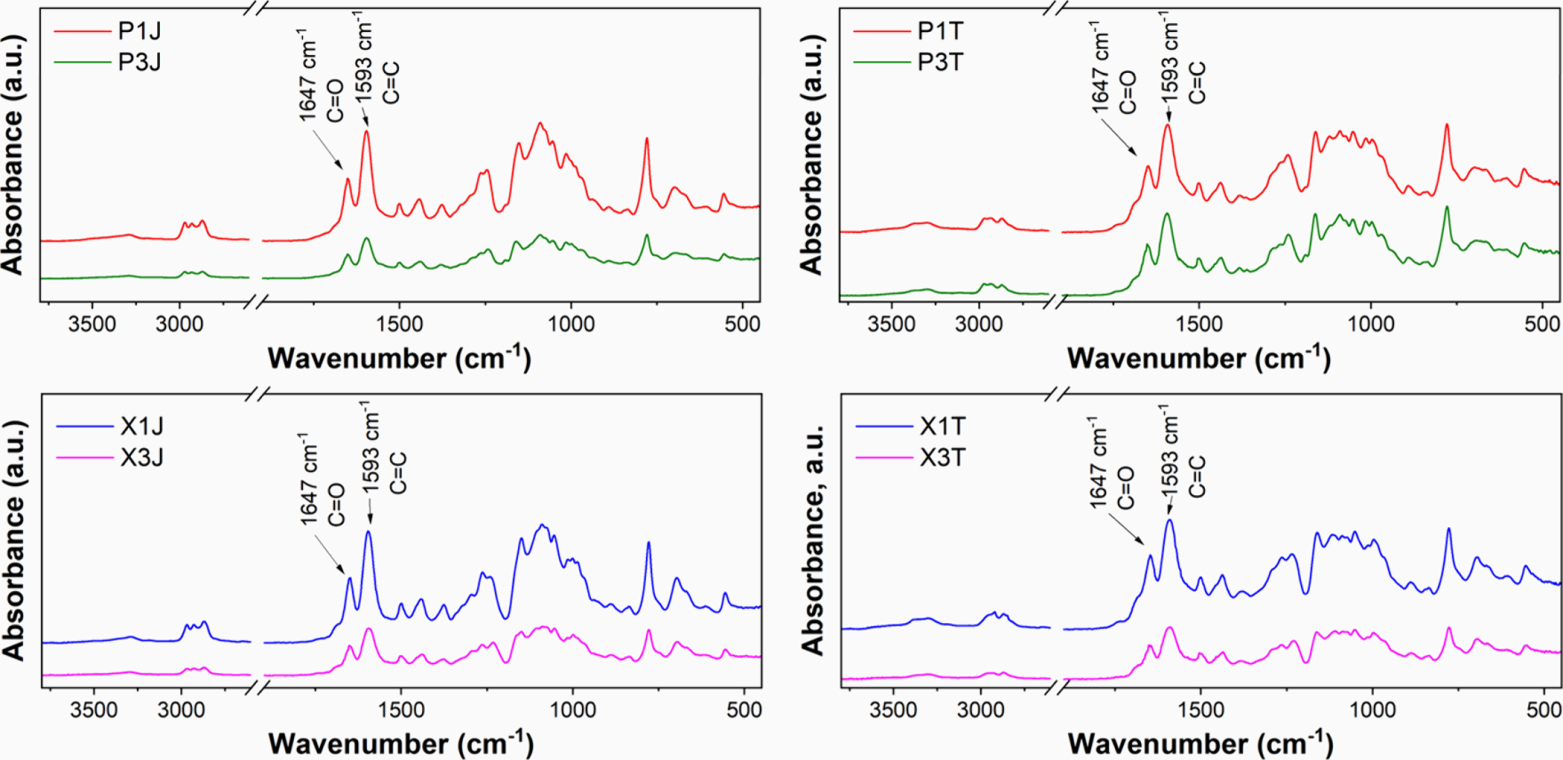
FTIR spectra of isosorbide-based CANs.

In total, a series of 8 polymer samples was prepared, differing in amine structure and the ratio of di- and triamines, which affected network density, chain rigidity, and final thermo-mechanical properties (Table 2).

**Table 2:** Thermomechanical Properties of Prepared CANs

sample ID	*T*_g_ *(°C)^a^*	*T*_α_(°C)^b^	*G*′_R_ (MPa)^c^	*Q*_m_	*w*_gel_ (wt %)	*T*_d5%_ (°C)
P1T	114	117	4.4	0.85 ± 0.02	98 ± 2	281
P3T	97	105	1.6	1.71 ± 0.05	95 ± 1	274
X1T	125	130	3.0	0.61 ± 0.02	99 ± 1	272
X3T	115	122	1.6	0.98 ± 0.01	96 ± 2	276
P1J	60	69	2.9	3.0 ± 0.1	81 ± 3	275
P3J	79	88	1.3	3.5 ± 0.1	86 ± 1	270
X1J	66	nd^d^	nd^d^	3.4 ± 0.1	90 ± 2	290
X3J	87	nd^d^	nd^d^	5.1 ± 0.1	83 ± 1	285

a From DSC (the 2nd heating run).

b From DMTA (the maximum value of tan δ curve).

c Storage modulus in the rubbery plateau at *T* = *T*_α_ + 50 °C.

d Too brittle sample for DMTA.

The DSC and DMTA results revealed the formation of glassy amorphous networks with a single main transition temperature (related to *T*_g_-DSC, or *T*_α_-DMTA) determined in the relatively broad range of *T*_g_ = 60–125 °C, respectively *T*_α_ = 69–130 °C (Table 2). These values were influenced by both cross-link density and chain rigidity (aromaticity). The DMTA results (Figure 3), showing the evolution of storage modulus (*G*′) and loss factor (tan δ), provided more detailed insight into the network arrangement. The values of storage modulus in the rubbery plateau (*G*′_R_) were then directly proportional to the cross-link density of CANs. In the case of CANs cured with the trifunctional amine TREN, the higher *T*_g_ (or *T*_α_) was primarily caused by the higher cross-link density, as also evidenced from the lower swelling degree (*Q*_m_, Table 2). Therefore, CANs with the higher TREN content (P1T and X1T) exhibited higher *G*′_R_ values compared to those of P3T and X3T (Table 2). Due to the presence of short aliphatic chains, CANs derived from TREN-branching units were glassy materials with a high *T*_g_. The addition of rigid aromatic but only bifunctional amines, PDFA or XYDI, led to the formation of CANs with a lower cross-link density and therefore a lower *T*_g_.

**Figure 3:**
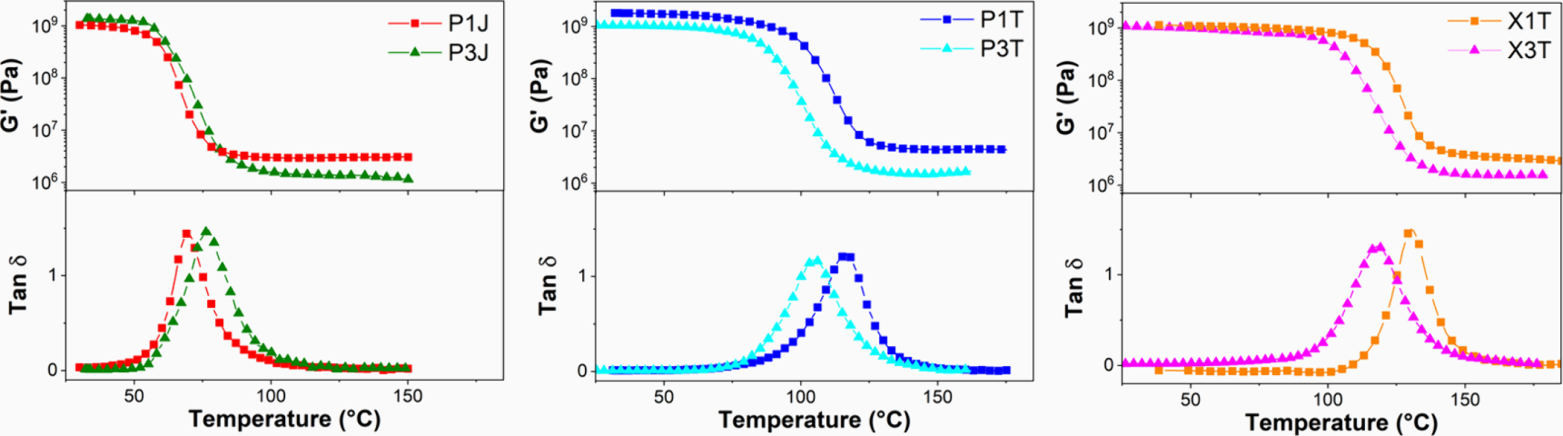
DMTA measurements of CANs: temperature dependence of storage modulus (*G*′) and loss factor (tan δ).

In contrast, the viscoelastic behavior of CANs based on the trifunctional amine JEFF was different (Figure 3), since the branching component of the system (JEFF) was composed of highly elastic polyoxypropylene chains. Thus, more elastic CANs were formed with *T*_g_ of 60–87 °C (Table 2). The increased content of bifunctional PDFA aromatic amine led to the formation of more dilute networks, as indicated by the higher *Q*_m_ values (Table 2). Surprisingly, the slightly higher *T*_g_ of P3J compared to P1J (Table 2) was observed due to the high rigidity of the aromatic PDFA structures in CANs, as well as the intensive interchain π-π interactions of the aromatic nuclei.^34^ The P3J sample was thus stiffer than P1J due to the higher content of incorporated aromatic PDFA, despite its lower cross-link density.

To determine the content of the cross-linked fraction, the gel content (*w*_gel_) of the prepared samples was determined by extraction in THF (Table 2). All CANs containing the trifunctional amine TREN (P1T, P3T, X1T, and X3T) exhibit a high gel content (>95%). In particular, the samples with a higher proportion of TREN cross-linker (P1T and X1T) showed a high gel content (>98%). This indicated an efficient cross-linking reaction and the formation of highly cross-linked materials with a low degree of swelling, which had a low content of sol components, not incorporated into the network structure. In contrast, the samples based on the trifunctional JEFF (P1J, P3J, X1J, and X3J) showed a lower amount of gel content (81–90%) and a high swelling ratio (3.0–5.1), indicating the formation of less cross-linked materials and networks with structural defects. It therefore seems that side reactions occur during cross-linking when the JEFF cross-linker is used. This may be due to the relatively long and flexible ether chains of JEFF, which increase the probability of ring formation caused by intramolecular reactions.^35,36^ This leads to the formation of macrocycles that are not chemically incorporated into the network structure of CANs and thus contribute to the sol fraction increase. In addition, the samples containing XYDI (X1J and X3J) were very brittle, which made their thermo-mechanical testing impossible, and after a longer swelling period, they disintegrated into small fragments.

The TGA results (Figures S7–S14) revealed that all prepared CANs displayed the *T*_d5%_ values in the range of 272–290 °C (Table 2), demonstrating their excellent thermal stability. This is achieved due to the synergistic effect of the high content of aromatic segments originating from ISO-AA and diamines and the high cross-link density of the produced CANs. Compared to previously reported vinylogous urethane CANs using only aliphatic amines,^27^ the thermal stability of the prepared CANs reported here is about 30–50 °C higher (Table 2). The thermal stability of CANs increased with the increasing cross-link density, regardless of the amine used.^29^

Once sufficiently thermally stable CANs were fabricated, their dynamic characters were evaluated using stress relaxation experiments, as bond exchange in CANs in the rubber state (above *T*_g_) releases stress proportional to the rate of this exchange.^37^ The relaxation modulus, *G*(*t*), was recorded as a function of time in the temperature range of 130–170 °C under constant strain of 4% (prior to the relaxation measurements, strain sweep experiments at 130 °C were conducted to determine LVE of the samples, Figure S15). The normalized relaxation modulus (*G*(*t*)/*G*_0_) of CANs decayed to its 1/*e* within a short time, on the order of tens to hundreds of seconds (Figure 4). Similarly, short relaxation times inducing rapid exchange transamination reactions between vinylogous urethanes and free NH_2_ groups were reported.^38^ However, it is worth emphasizing that such short relaxation times were herein achieved without the use of any external catalysts. Full relaxation was achieved for P1T and X1T at every tested temperature (Figure 4). In contrast, for P3T and X3T samples (Figure 4), no complete relaxation was observed within a reasonable time frame (after a prolonged measurement period, a certain degree of thermal degradation was observed, especially in the case of X3T). The partial degradation for X3T at elevated temperatures can be attributed to its lower cross-link density and less homogeneous network structure. It therefore seems that a higher proportion of TREN units in the CAN structure is necessary for complete relaxation and efficient exchange.

**Figure 4:**
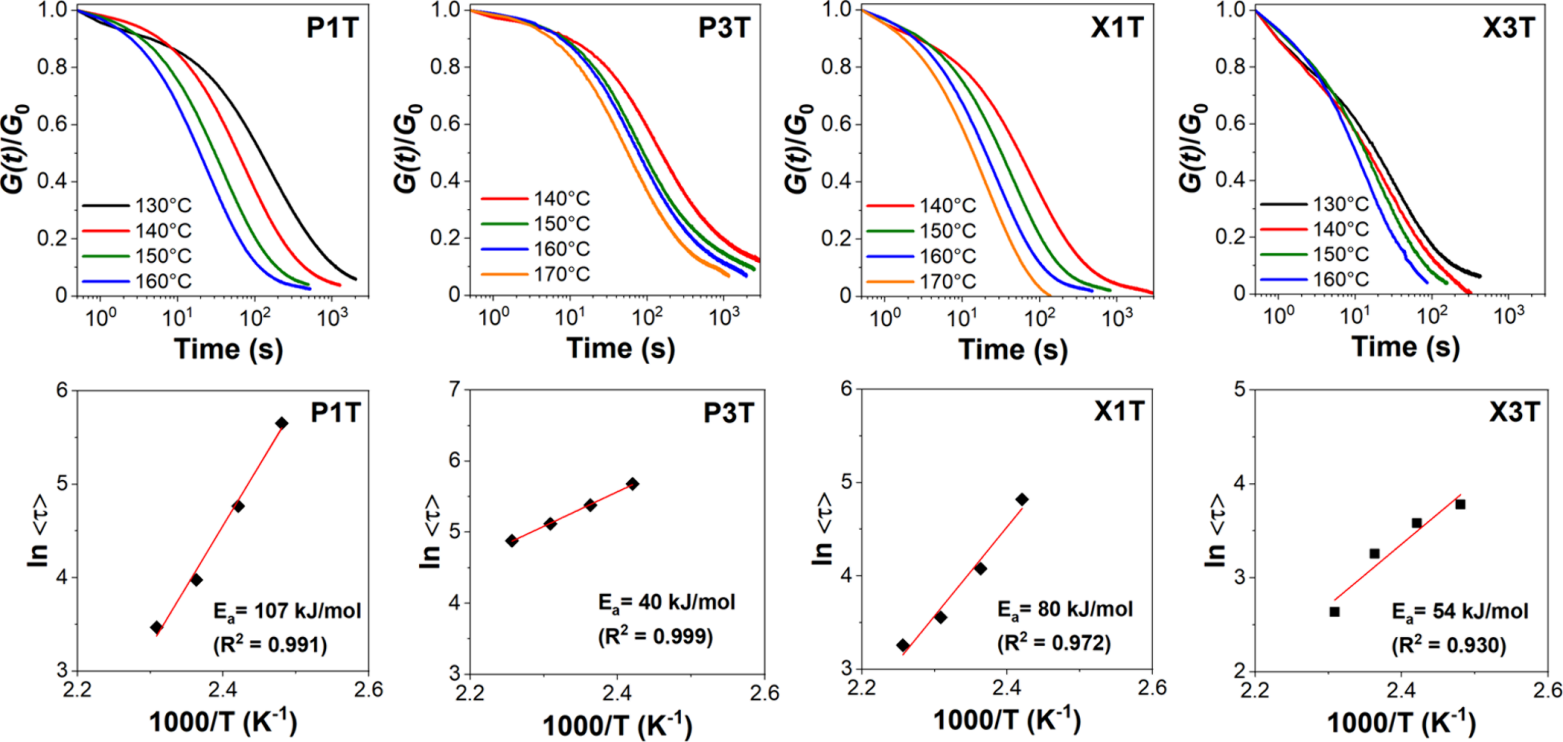
Normalized stress relaxation curves and Arrhenius plot for CANs (original stretch relaxation curves were fitted using Kohlrausch–Williams–Watts stretched exponential decay function to determine the characteristic average relaxation time, ⟨τ⟩).

To further quantify the dynamic behavior, the stress relaxation curves of CANs were fitted. The first fitting using the single-mode exponential decay Maxwell model (eq 1) was found to be inappropriate (Figure S16 and Table S1). It is known that the stress relaxation curves of the vinylogous CANs do not correspond to simple exponential decays and show a complex relaxation mode, due to trapped loops, dangling chains, and associative interaction between polymeric chains.^39,40^ Therefore, the Kohlrausch–Williams–Watts (KWW) stretched exponential decay function (eq 2) was used to fit the CAN stress relaxation curves, which led to much better results (Figure S17). The average relaxation times, ⟨τ⟩, calculated according to eq 3, and fitting parameters (τ*, β) as functions of temperature are summarized in Table S2. The β stretch exponent is a shape parameter characterizing the width of the relaxation distribution (narrower relaxation distribution, higher β values). The β value herein for all CANs ranged within a range of 0.60–0.80 and increased slightly with increasing temperature (Table S2), which corresponds well to data from the literature.^41^ The determined β values can be associated with the formation of a segmentally heterogeneous network reflecting different relaxation dynamics due to the presence of structurally different amines.^42^ The average relaxation time, ⟨τ⟩, decreased with the temperature increase according to Arrhenius law for P1T, P3T and X1T, which enabled us to calculate stress relaxation activation energy (*E*_a_, Figure 4).^33^ In the case of X3T, the Arrhenius fit showed a lower *R* ^2^ value of 0.93, probably due to incomplete relaxation and partial thermal degradation of the sample during the relaxation measurements.

The relaxation behavior of prepared CANs showed dependence on the diamine/triamine ratio. The higher content of trifunctional TREN (P1T and X1T samples) apparently promoted faster and complete relaxation of CANs, independent of the type of diamine used. In the case of P3T, slower relaxation was observed in the temperature range 140–170 °C (Figure 4), and at 130 °C full relaxation was not achieved (Figure S18). Moreover for P1T and X1T systems, the strong temperature-dependence of the ⟨τ⟩ values was observed compared to the weakly *T*-dependent ⟨τ⟩ values of P3T and X3T. As a consequence, the *E*_a_ values (calculated from the slope) of the P1T (107 kJ/mol) and X1T (80 kJ/mol) systems are higher compared to those of the P3T (40 kJ/mol) and X3T (54 kJ/mol) systems. The lower *E*_a_ values due to the decreased slope in the Arrhenius plot imply a more significant rate of exchange reactions at lower temperatures.^43^ However, all *E*_a_ values reported here are very close to the values mentioned for other vinylogous urethane vitrimers.^29^ The *E*_a_ values of the highly cross-linked CANs (P1T and X1T) were found to be significantly higher than that of the lowered cross-linked P3T and X3T (see the *G*′_R_ values directly corresponding to the cross-link density in Table 2). A similar dependence of activation energy on cross-link density was previously observed for polyimine vitrimers.^44^

### Material Recycling/Reprocessing

A characteristic feature of vinylogous urethane CANs is their recyclability via reprocessing due to reversible thermally triggered transamination of vinylogous urethane bonds occurring in the network (Scheme 1).^37^ To investigate the reprocessability of the prepared CANs, P1T and X1T samples were selected as the most promising CANs for potential replacement of tradition epoxy networks, due to their (i) high cross-link density and rigidity resulting in high *T*_g_ (Table 2), (ii) mechanical robustness resulting in high tensile stiffness and strength while keeping sufficient toughness (Figure S19), and (iii) fast exchange transamination (Figure 4).

The P1T and X1T samples were ground into powder and reprocessed again by compression molding. The stress–strain curves of original (virgin) and recycled CANs are very similar (Figures S20–S21). Tensile tests thus show the preservation of the mechanical properties of the prepared CANs after reprocessing (Figure 5). All changes in mechanical properties were statistically insignificant, except for the tensile stress at break of X1T, which even increased by 44% after reprocessing. This slight increase in tensile strength can be the result of more homogeneous reorganization of the network during reprocessing.^27^

**Figure 5:**
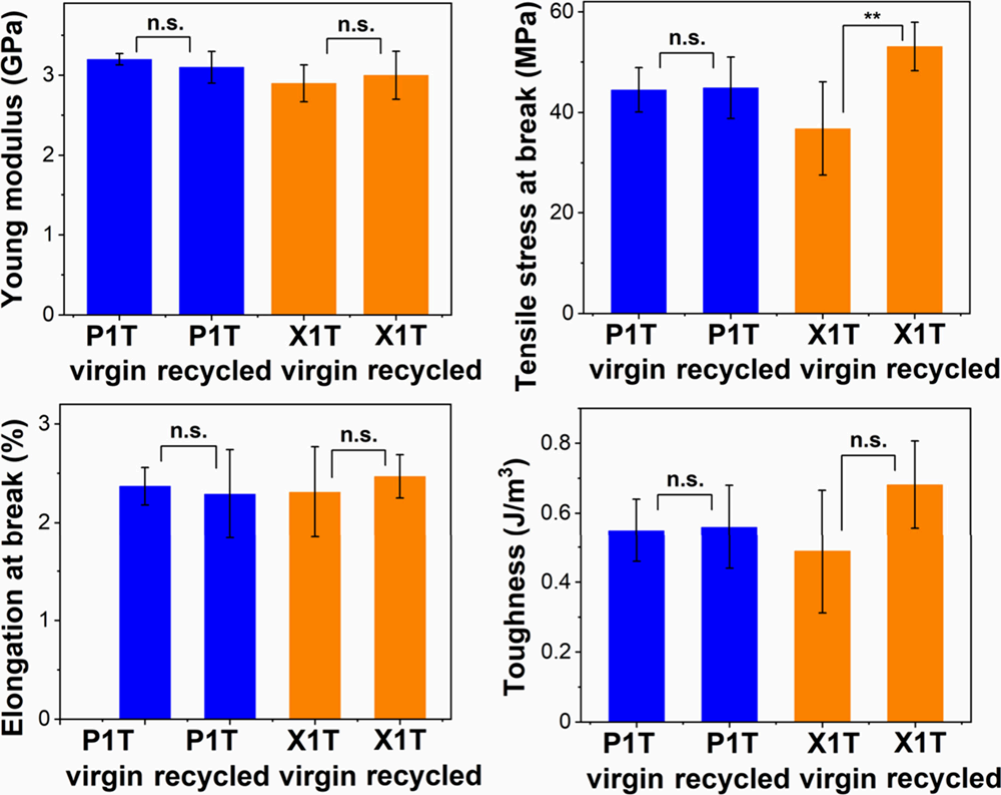
Young modulus, tensile stress at break, elongation at break, and toughness of CANs before (virgin) and after (recycled) reprocessing. Error bars represent standard deviation (*n* > 6). Tukey test: **p < 0.01, ***p < 0.001, n.s., not significant.

The DSC results show that the *T*_g_ values of the recycled CANs remained almost unchanged, indicating that the cross-link density of the materials was preserved (Figure 6). It proves that reprocessing occurred via associative bond exchange, rearranging the network topology without reducing cross-link density.^28^ A slight *T*_g_ reduction in the case of P1T may indicate incomplete recross-linking or side reactions promoting chain scission. However, this backbone cleavage had to proceed in minor extent since the mechanical properties were not negatively influenced (Figure 5).

**Figure 6:**
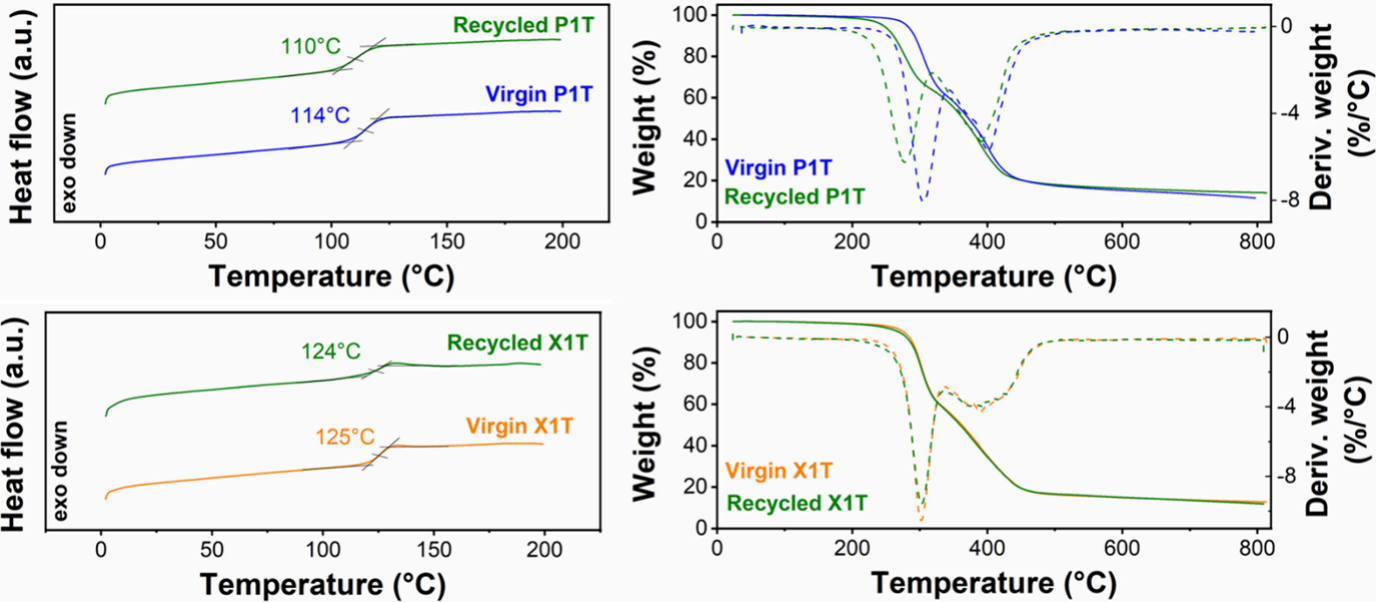
DSC traces (left) and TGA curves (right) of CANs before (virgin) and after (recycled) reprocessing. The temperature values on DSC curves indicate the glass transition temperature (*T*_g_) determined as the inflection point during the second heating run at 10 °C/min. TGA results show weight loss (solid lines) and derivative weight loss (dashed lines) curves during heating at 10 °C/min under nitrogen flow.

TGA results show how the reprocessing affects the thermal stability of CANs (Figure 6). While the thermal stability of X1T practically did not change after recycling, the thermal stability of the reprocessed P1T sample deteriorates (*T*_d5%_ decreased from 284 to 245 °C), indicating a certain degree of thermally induced degradation. This can be caused by impurities as a result of the difficult synthesis of PDFA, which, despite improvement compared to other studies, still leads to relatively low yields and difficult product purification.

### Comparison with Other Vitrimeric Systems

In this paper, the fabricated CANs are developed as a potential replacement for conventional epoxy thermosets used for engineering composites (e.g., GFRP and CFRP laminates in wind turbine blades, automotive, marine, and other transport and construction sectors).^45^ For these structural-grade thermosets, a *T*_g_ > 120 °C is a fundamental requirement to maintain stiffness and prevent softening during the product life.^46^ Table 3 lists the *T*_g_ of glassy vinylogous urethane vitrimers that have been mentioned in scientific studies in recent years. It can be seen that the *T*_g_ of most materials is below 90 °C, and reaching temperatures >120 °C is still a great challenge. However, in this study, we were able to prepare mechanically robust vitrimers P1T and X1T with *T*_g_ (from DMA) of 117 and 130 °C, respectively, thanks to a suitable combination of high stiffness, aromatic (benzene and furan core) components, and increased cross-link density. In addition, we managed to incorporate a high level of biobased components in the P1T material. In further research, we will focus on studying the wettability and adhesion with selected fibers in order to verify the applicability of the developed CANs for the preparation of structural composites.

**Table 3:** Glass Transition Temperatures (*T*_g_) of Glassy Vinylogous Urethane Vitrimers Reported in the Literature

vitrimer structure	*T*_g_–DSC^a^ (°C)	*T*_g_–DMA^b^ (°C)	ref
isosorbide	114, 125	117, 130	our study
isosorbide	36–57		^27^
PETG		80–83	^47^
polystyrene		53–76	^48^
isohexides		20–63	^29^
vanillin	50–85		^34^
cyclohexane-based	87		^6^
diamide-based	55–72		^40^
lignin		85–87	^49^

a Glass transition temperature obtained from DSC – the second heating at a rate of 10 °C/min.

b Glass transition temperature determined as the maximum of *tan* δ peak at a rate of 3 °C/min.

## Conclusions

In this work, we have developed a novel class of rigid biobased vinylogous urethane covalent adaptable networks (vitrimers) by combining d-isosorbide bis­(acetoacetate) with a furfural-derived diamine (PDFA). Both of these monomers contain a high amount of renewable carbon in their structures. Such monomer design enables simultaneous enhancement of renewable carbon content and network rigidity, resulting in glassy vitrimers with high thermal stability and tunable thermomechanical properties. The obtained materials were prepared via a solvent-free bulk curing strategy, demonstrating a scalable and environmentally favorable approach to cross-linked polymer networks. Depending on the structure and ratios of amine monomers, the resulting vitrimers showed glass transition temperatures up to 125 °C and thermal stability approaching 290 °C.

The dynamic nature of the vinylogous urethane bonds enabled fast, catalyst-free stress relaxation in the viscoelastic state, with relaxation times in the order of tens to hundreds of seconds in the temperature range of 130 – 170 °C, and activation energies depending on the diamine/triamine ratio. Finally, our results show that the mechanical strength of the prepared CANs did not significantly deteriorate after the thermal recycling cycle, confirming that the amine-exchange reactions efficiently restore the cross-linked network after processing. The combination of high *T*_g_, mechanical robustness, and reprocessability places these polymer materials among the rare examples of rigid biobased vitrimers suitable for structural composite applications. Overall, this work establishes a clear molecular design strategy for high-performance, high-*T*_g_ sustainable vitrimers and provides a foundation for further development toward fully biobased, recyclable thermosetting materials.

## Supplementary Material



## Notes

Additional characterization of monomers by NMR and FTIR, additional characterization of vitrimers by FTIR, TGA, tensile curves, additional data on vitrimer relaxation and recycling (PDF)
