# Supplementary material for: Rigid Biobased Vinylogous Urethane Vitrimers from d‑isosorbide/Furfural-Derived Monomers
Source: ACS Polym Au. 2026 Jun 19;6(4):1147–56. doi: 10.1021/acspolymersau.6c00063 (PMC13474345; doi:10.1021/acspolymersau.6c00063)
Supplement: Supplementary file 1 [file lg6c00063_si_001.pdf]

Supporting information

Rigid Biobased Vinylogous Urethane Vitrimers from *D*-isosorbide/Furfural-derived Monomers

Ondrej Kopilec,[a] *Jiri Hodan,*[b] *Ondrej Sedlacek,*[a,*] *Hynek Beneš,*[b,*]

[a] Department of Physical and Macromolecular Chemistry, Faculty of Science, Charles University, 128 00 Prague 2, Czech Republic

[b] Institute of Macromolecular Chemistry of the Czech Academy of Sciences, 162 00 Prague 6, Czech Republic

E-mails: sedlacek@natur.cuni.cz, benesh@imc.cas.cz

Number of Pages: 18

Number of Schemes: 01

Number of Figures: 21

Number of Tables: 02

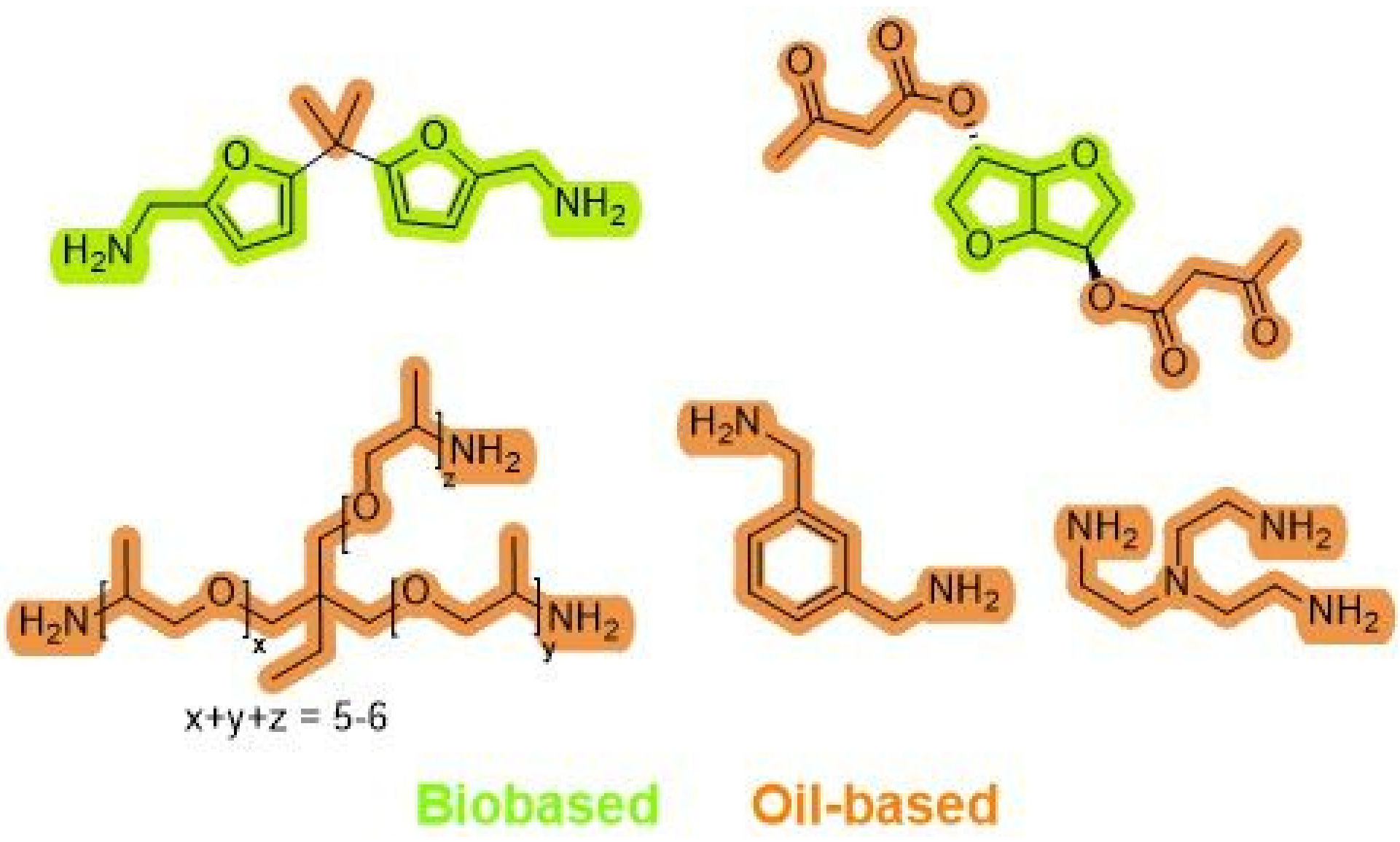

Scheme S1. Structures of monomers used for CAN synthesis highlighting their biobased and oil-based origin. Acetone, used in the synthesis of PDFA, was not counted as biobased, even though the Weizmann process can produce biobased acetone [1]. Similarly, although there is evidence that XYDI can be synthesized from biobased furfural [2], it was treated as an oil-based chemical in this study.

[1] Nguyen, N. P. T., Raynaud, C., Meynial-Salles, I. & Soucaille, P. Reviving the Weizmann process for commercial n-butanol production. Nature Communications 9, (2018).

[2] Scodeller, I. et al. Synthesis of Renewable meta-Xylylenediamine from Biomass-Derived Furfural. Angewandte Chemie - International Edition 57, 10510–10514 (2018).

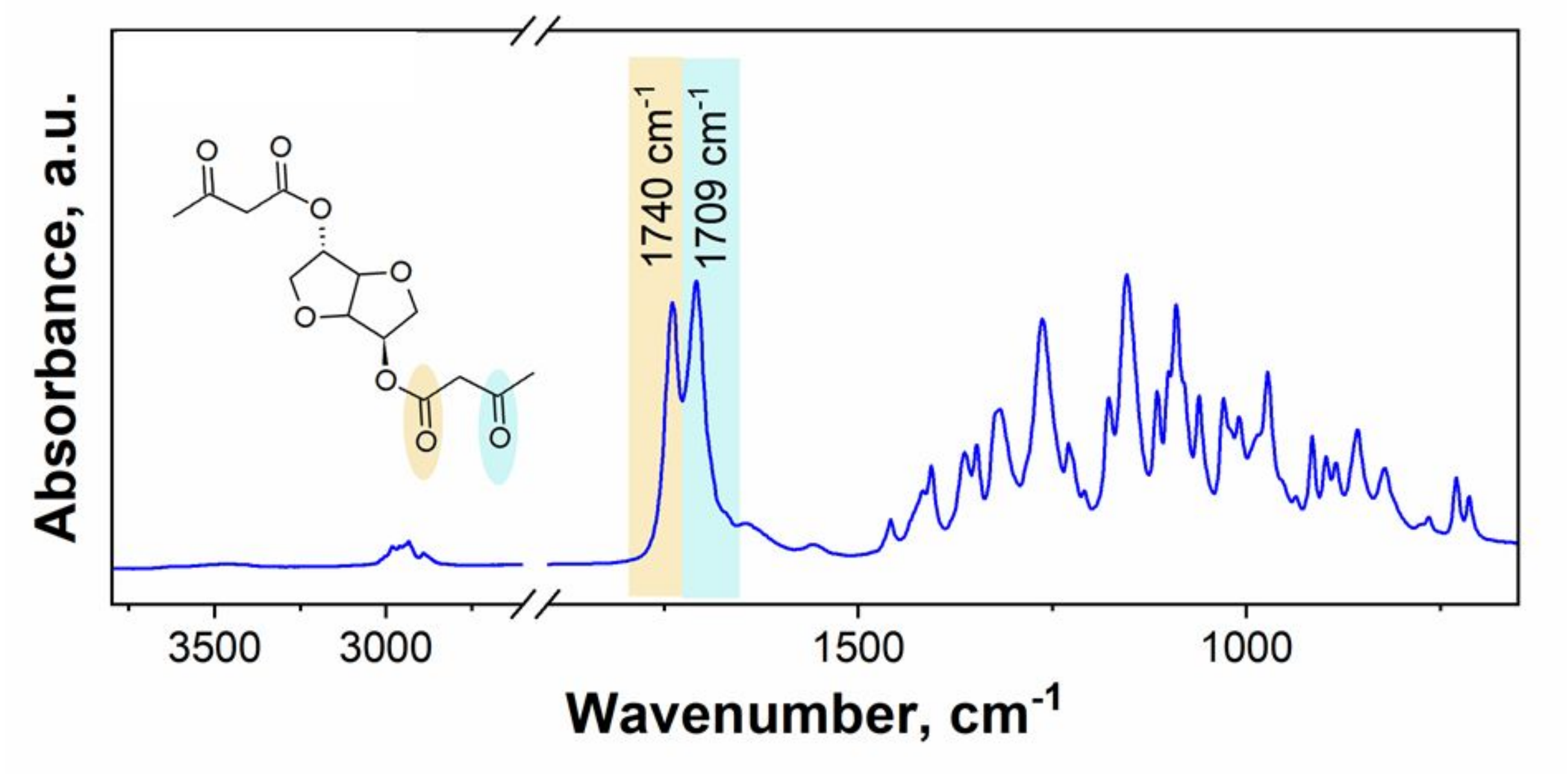

Figure S1. FTIR spectrum of ISO-AA with highlighted ester (1740 cm^{-1}) and ketone (1709 cm^{-1}) bands.

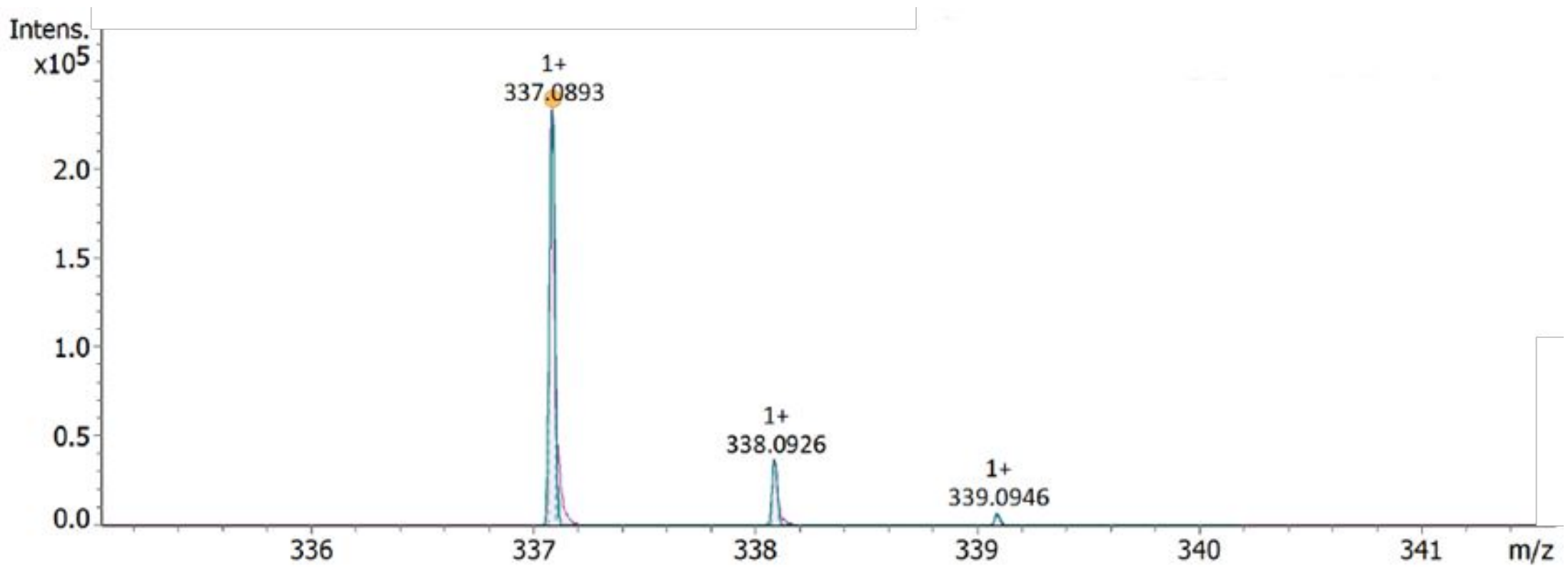

Figure S2. ESI-MS spectrum of ISO-AA

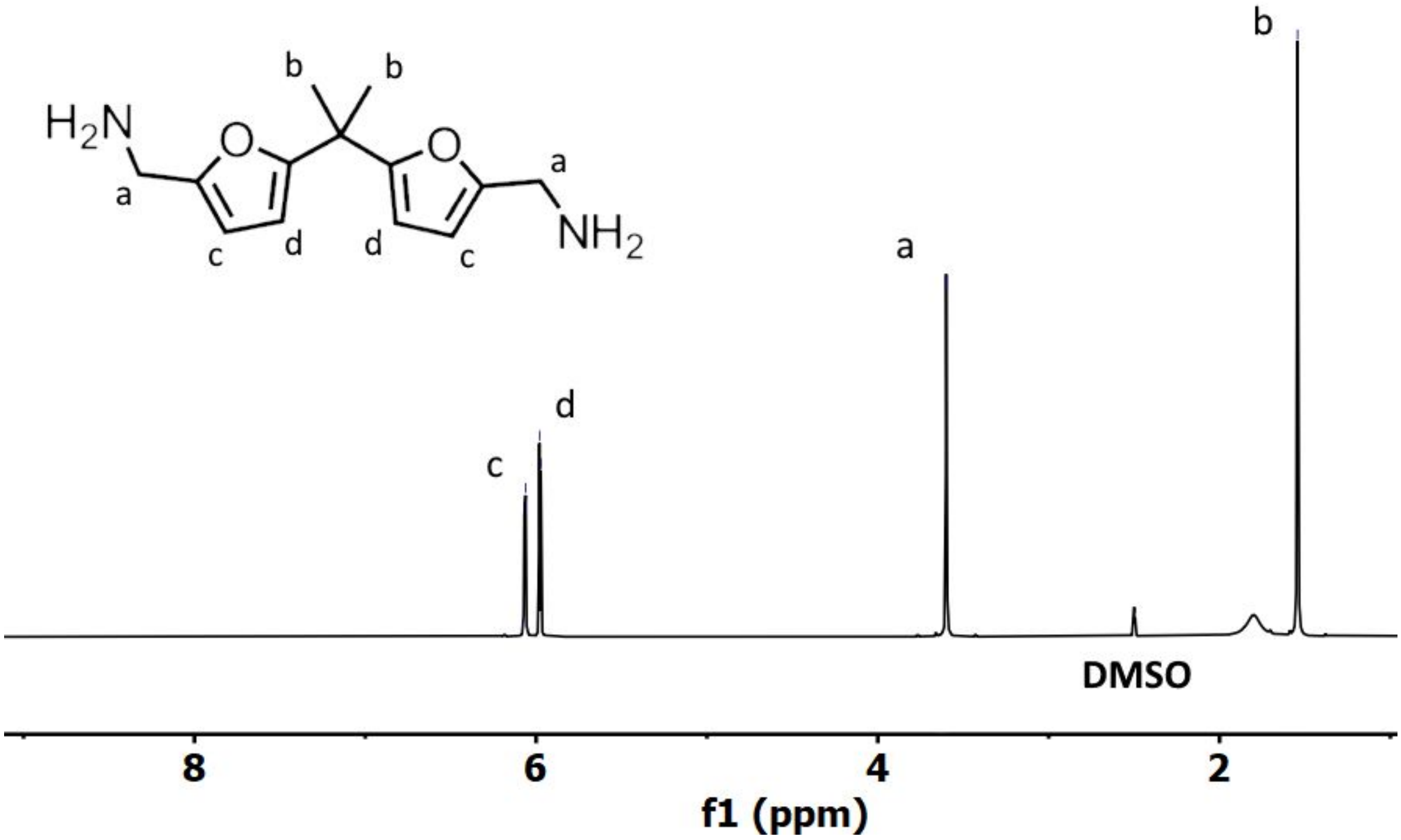

Figure S3. ^{1}H NMR spectrum of PDFA in DMSO-d_6.

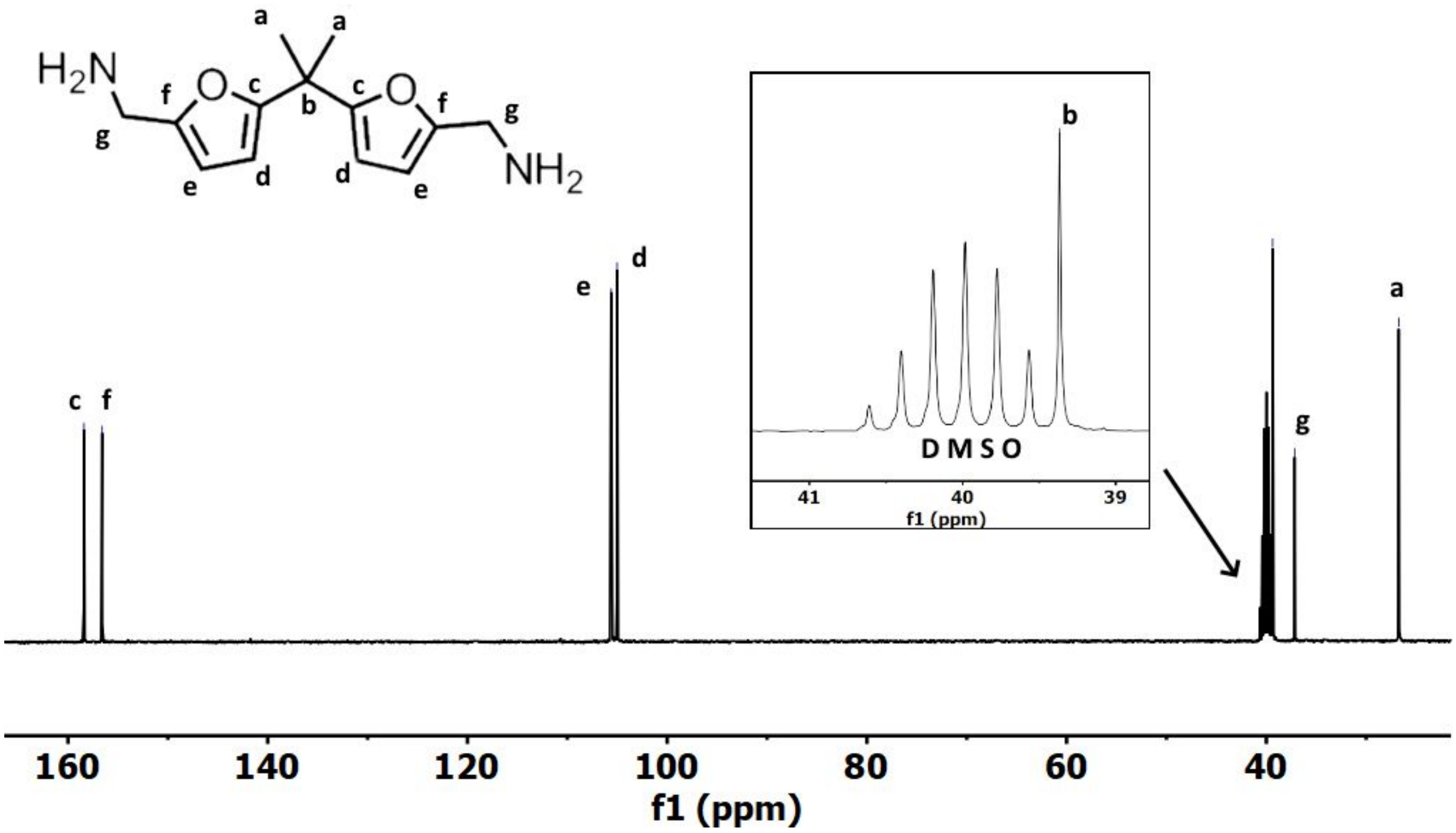

Figure S4. ^{13}C NMR spectrum of PDFA in DMSO-d_6.

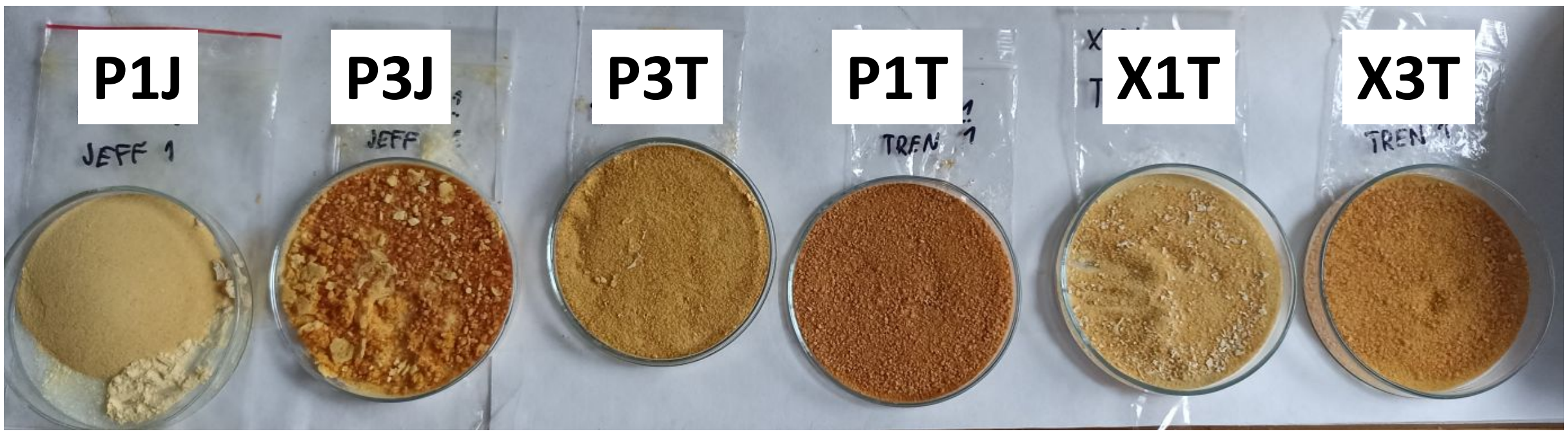

Figure S5. Prepared CANs after grinding.

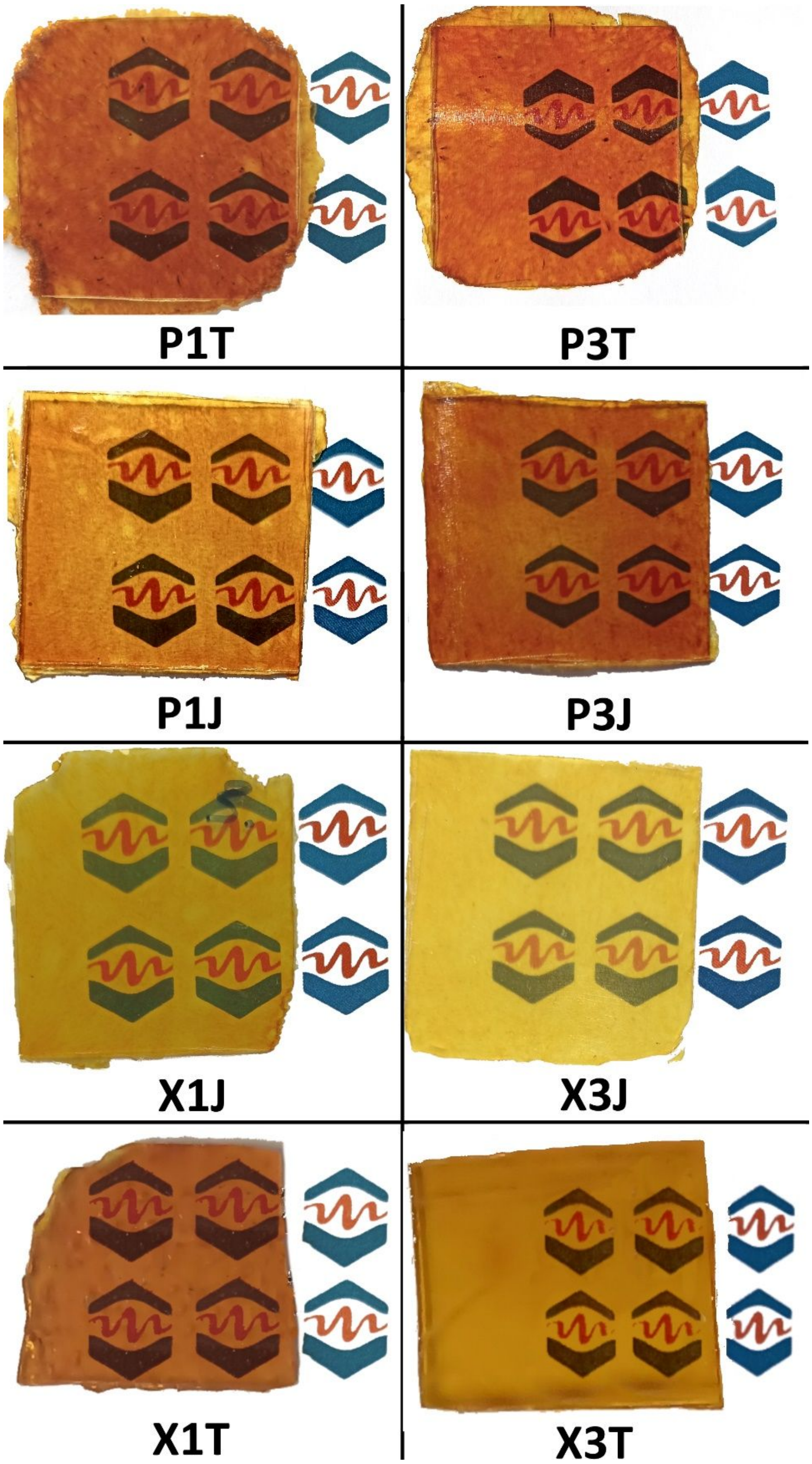

Figure S6. Photos of the prepared CANs (0.7 mm thick plates) after compression molding at different conditions.

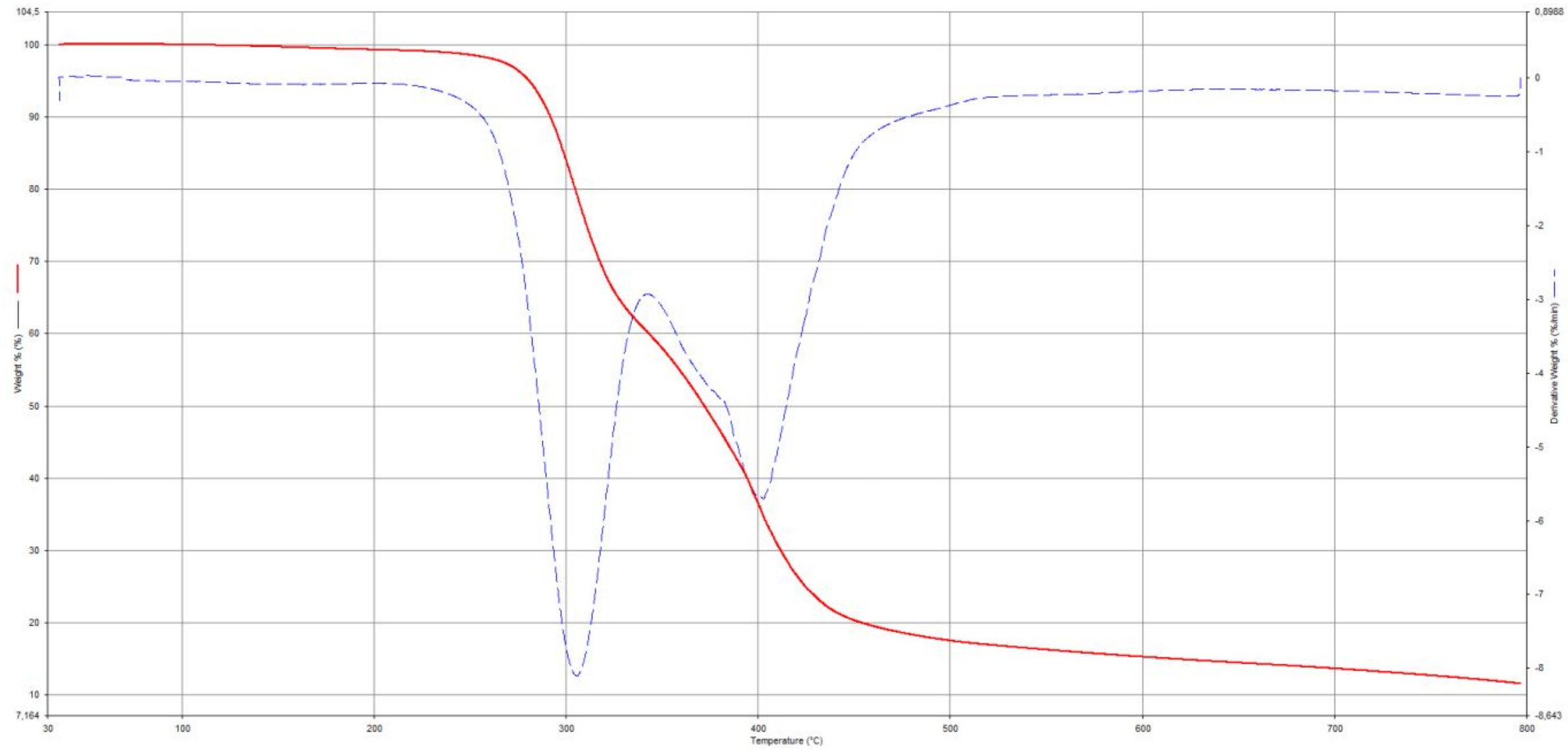

Figure S7. TGA and DTG curves of P1T.

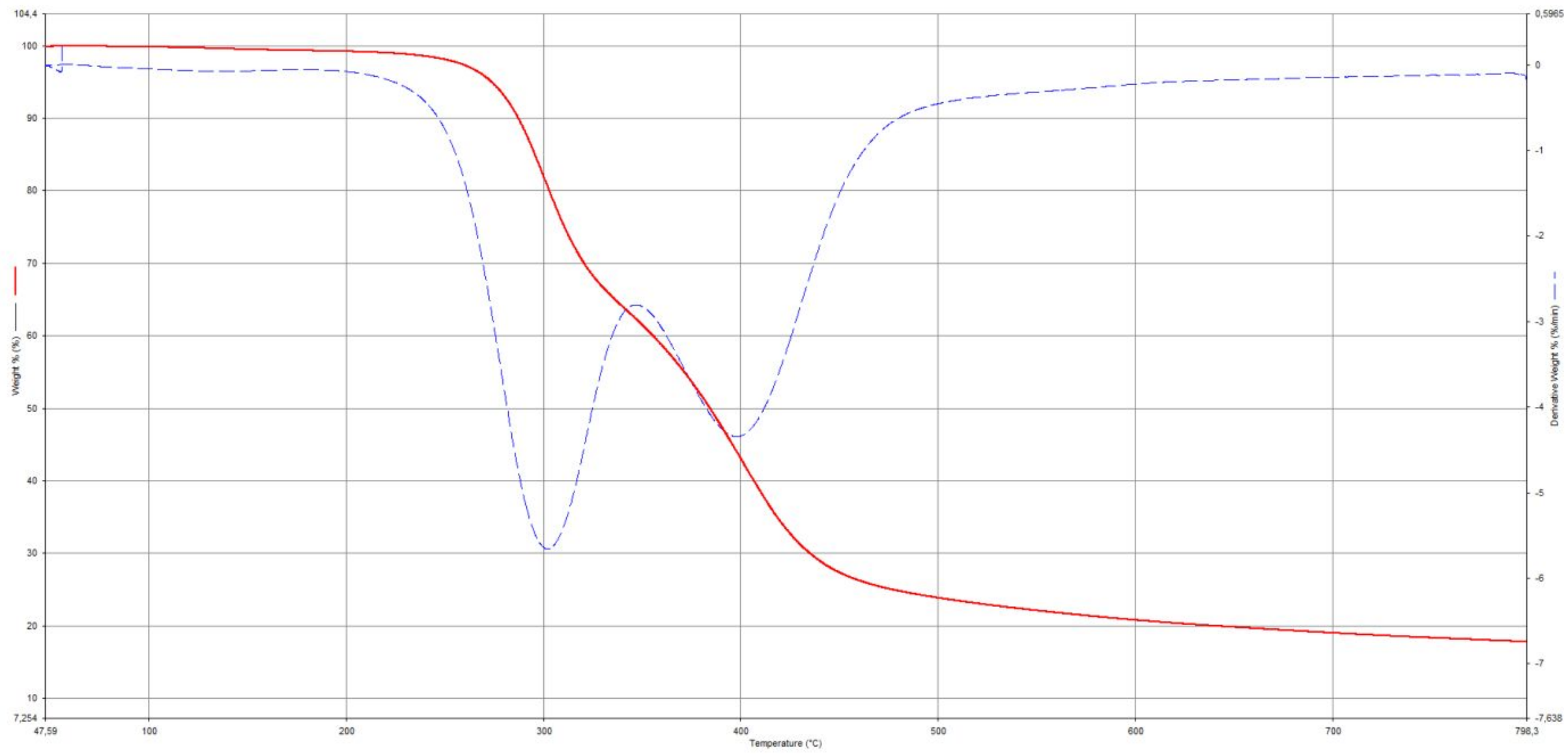

Figure S8. TGA and DTG curves of P3T.

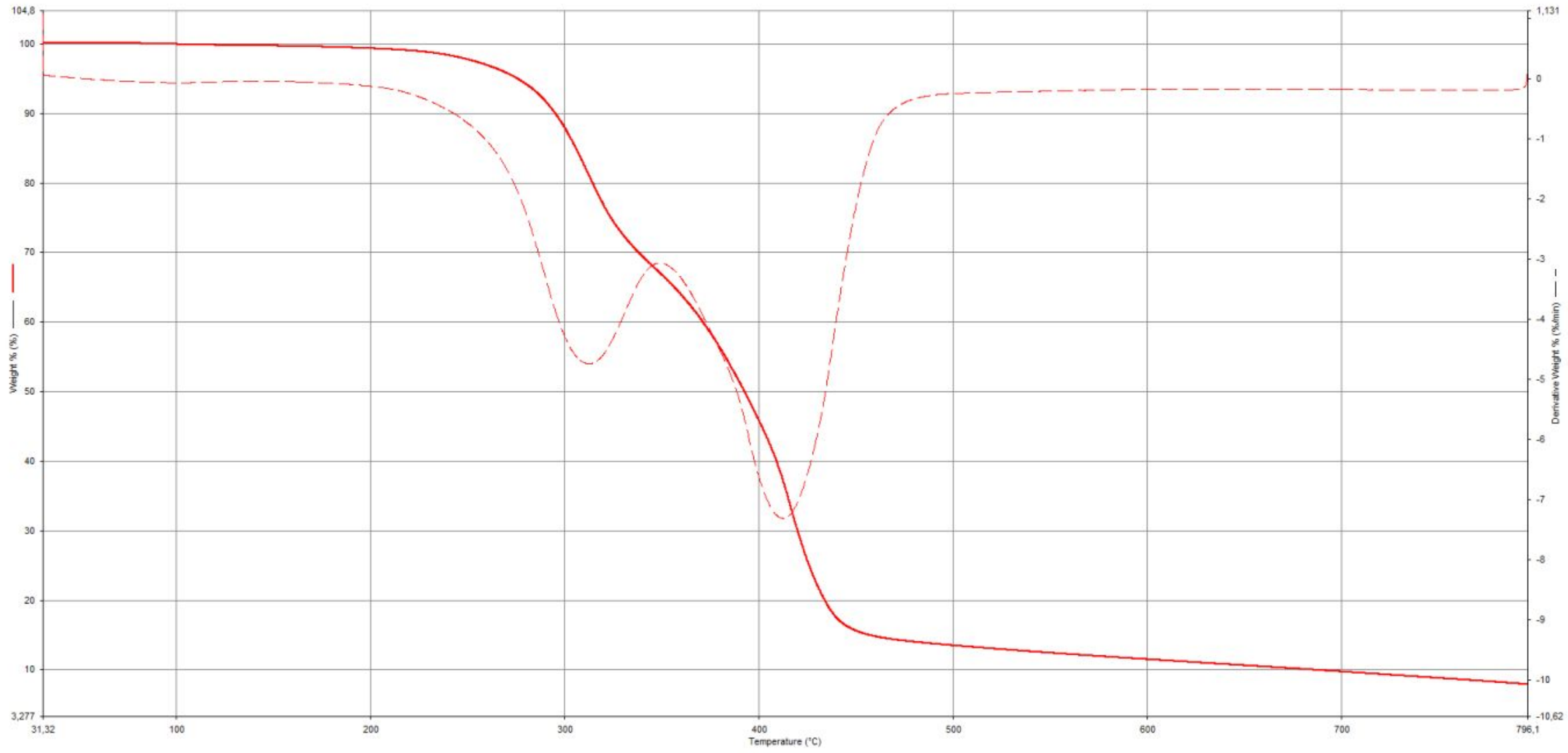

Figure S9. TGA and DTG curves of P1J.

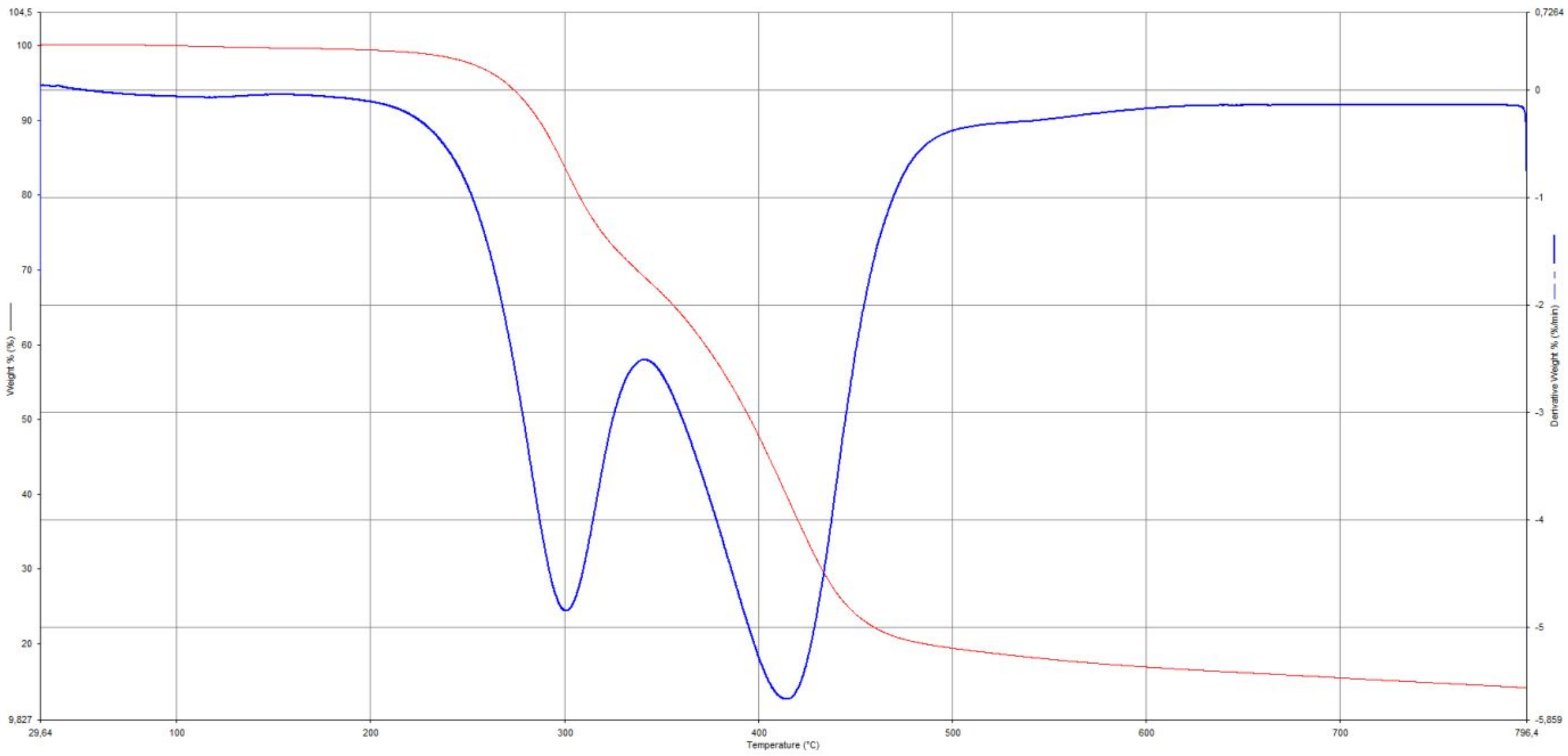

Figure S10. TGA and DTG curves of P3J.

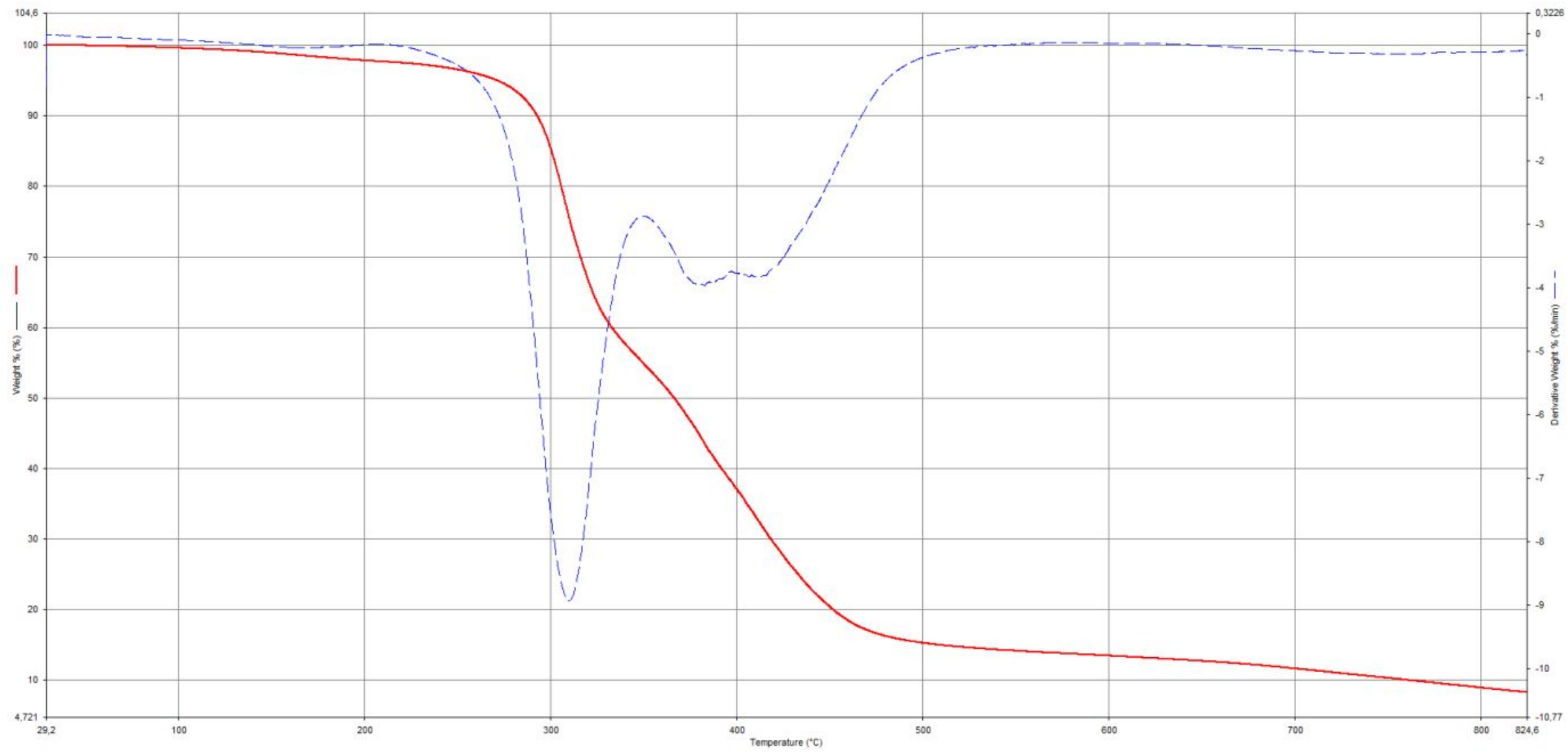

Figure S11. TGA and DTG curves of X1T.

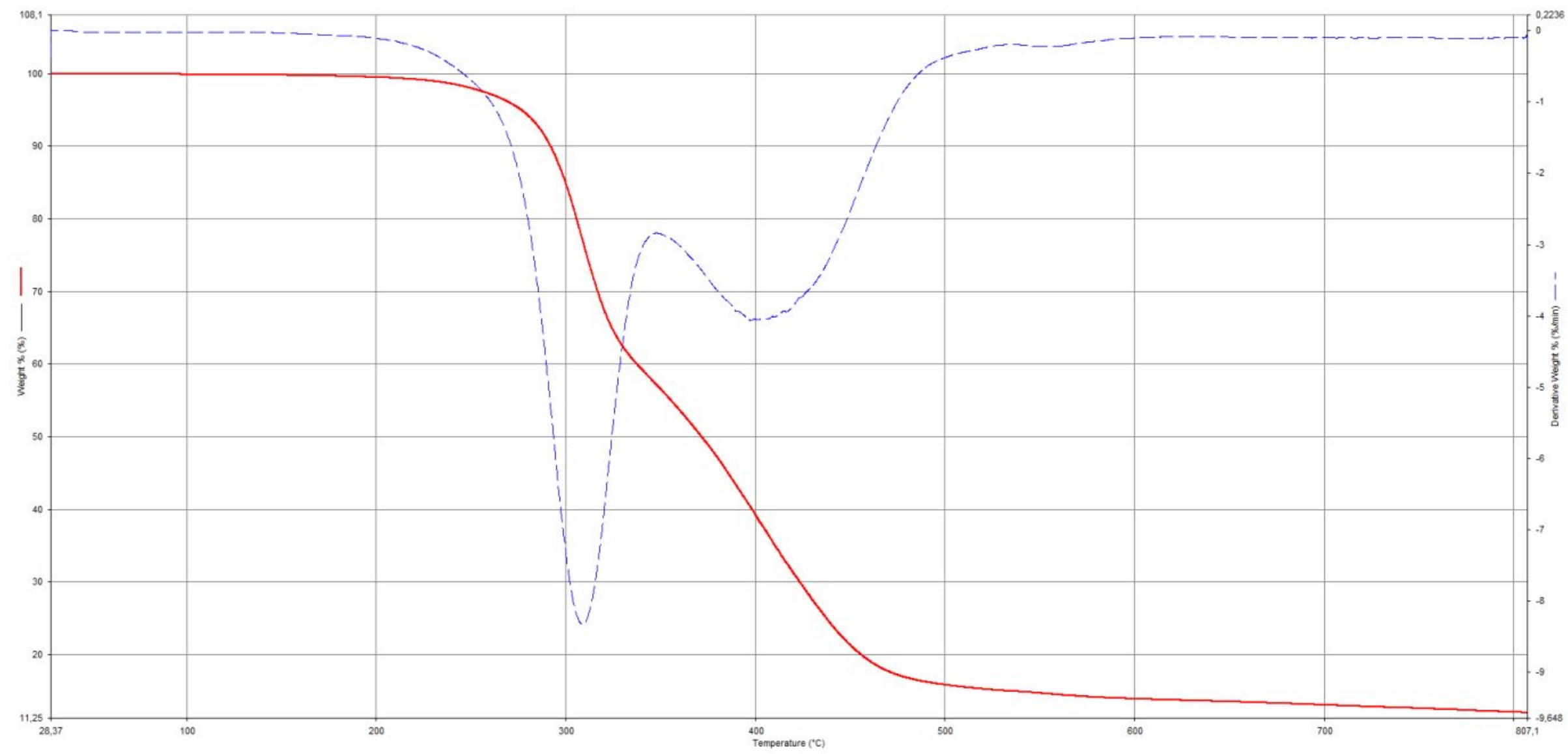

Figure S12. TGA and DTG curves of X3T.

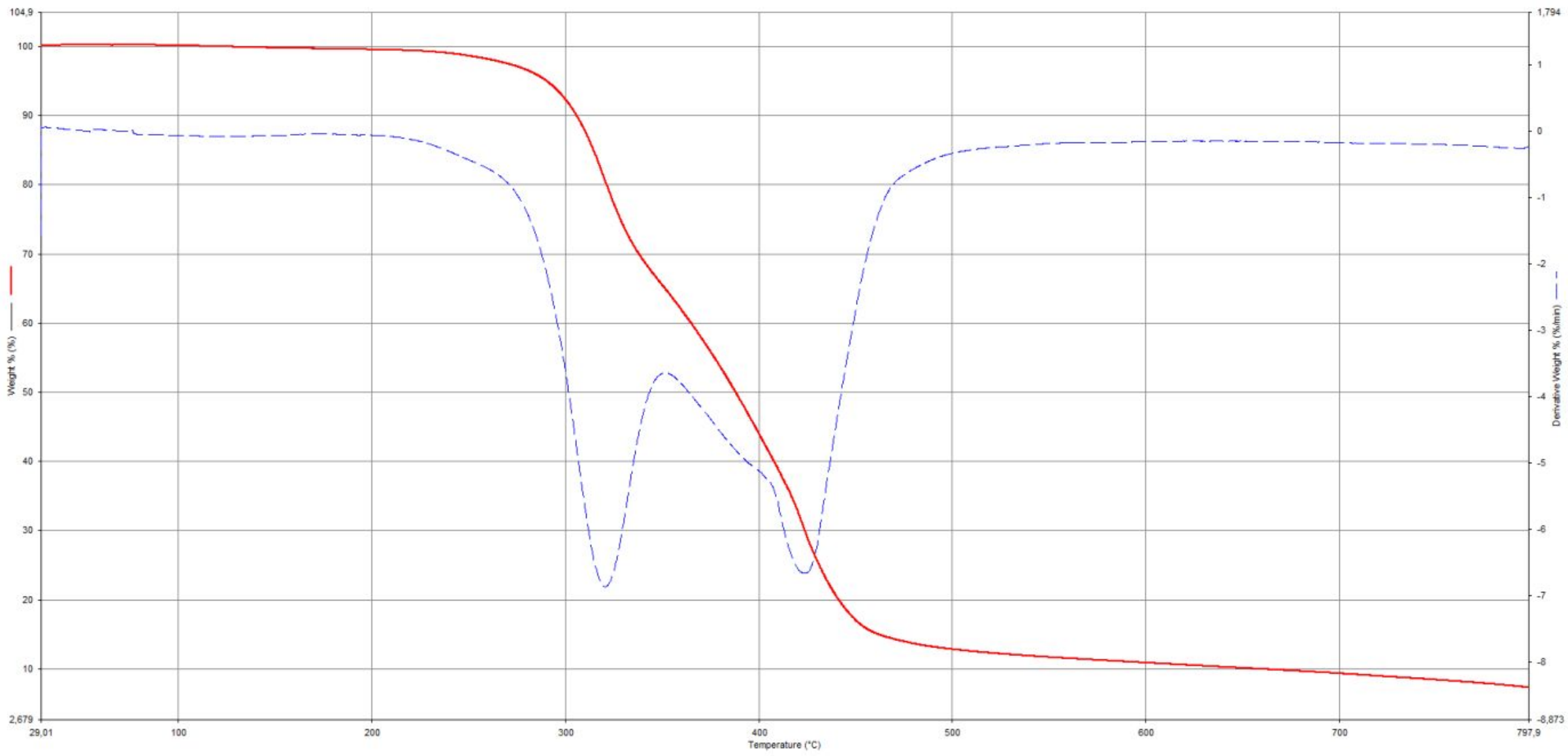

Figure S13. TGA and DTG curves of X1J.

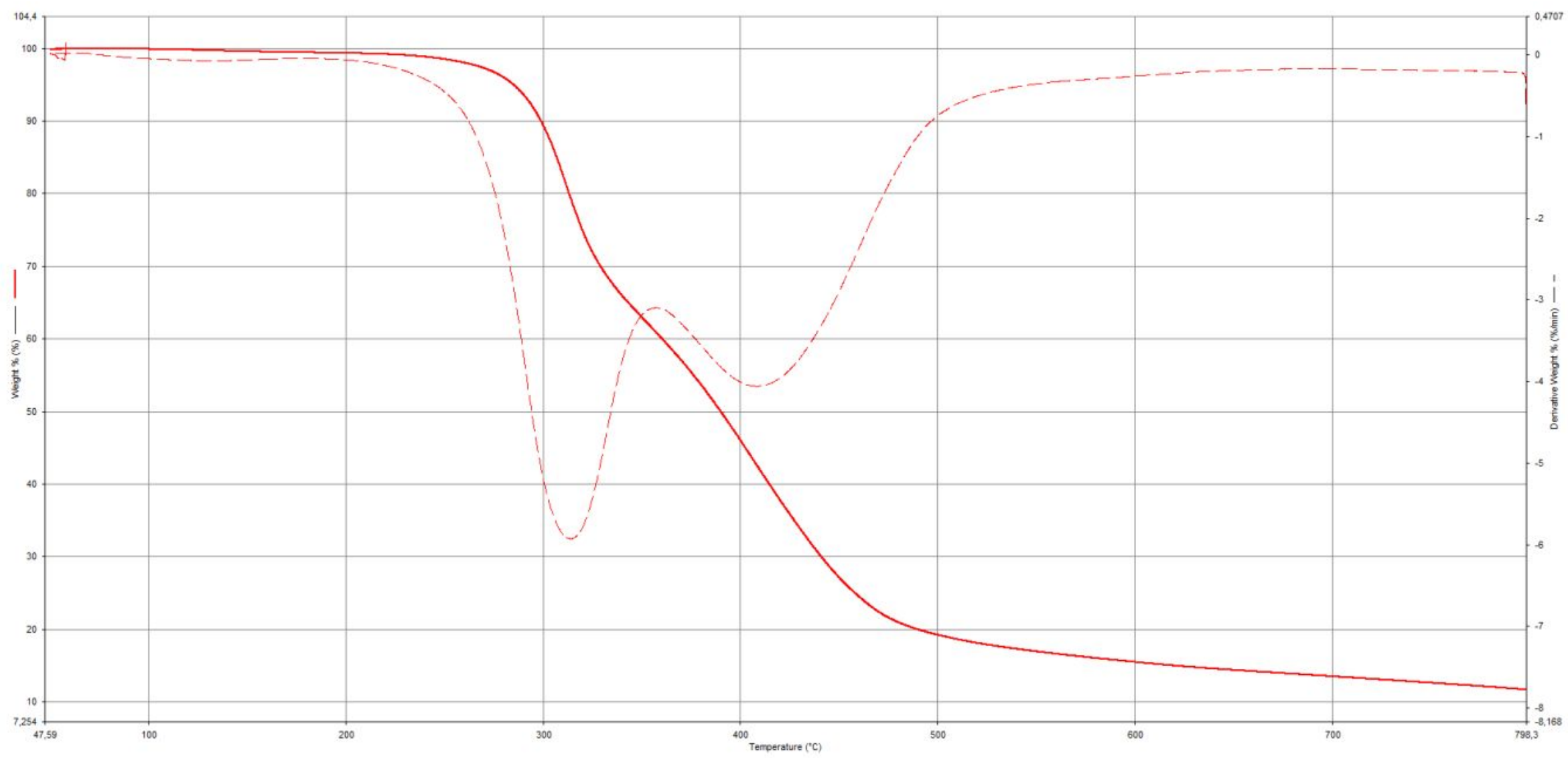

Figure S14. TGA and DTG curves of X3J.

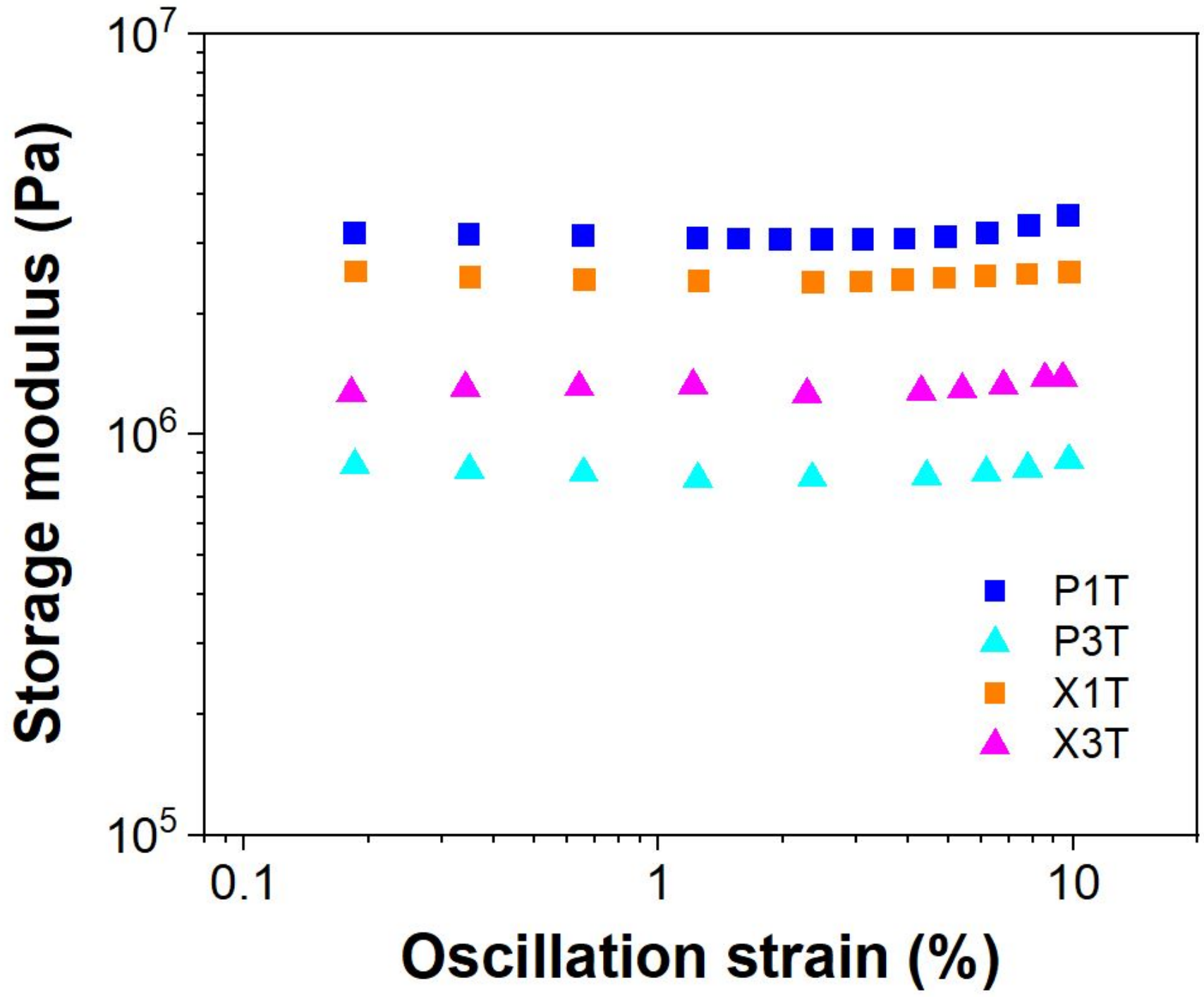

Figure S15. Strain sweep curves of CANs at 130 °C showing LVE region.

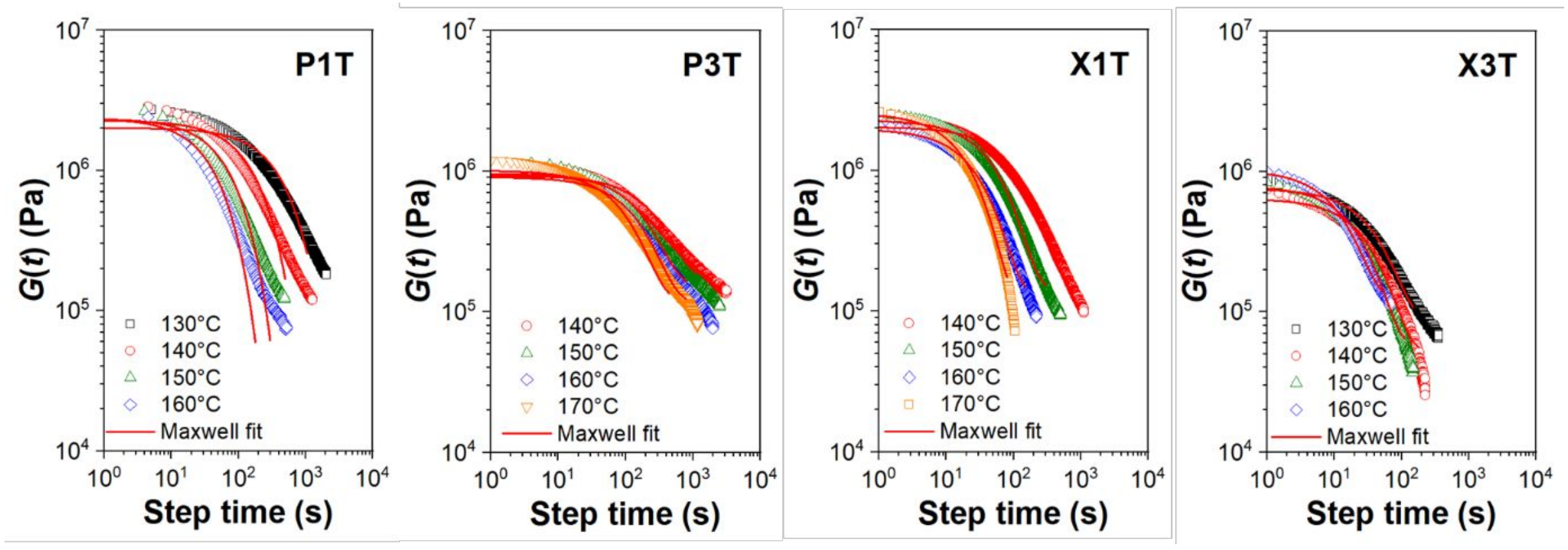

Figure S16. Stress relaxation curves of CANs at different temperatures and the results of fitting according to the single-mode exponential decay Maxwell model.

Table S1. Results of stress relaxation curves fitting according to the single-mode exponential decay Maxwell model.

Sample ID	Temperature (°C)	Maxwell	
		τ^* (s)	R^2
P1T	130	508	0.914
	140	191	0.918
	150	82	0.940
	160	49	0.938
P3T	140	304	0.971
	150	196	0.957
	160	168	0.964
	170	110	0.977
X1T	140	125	0.985
	150	60	0.991
	160	34	0.993
	170	23	0.995
X3T	130	44	0.984
	140	37	0.980
	150	25	0.990
	160	14	0.997

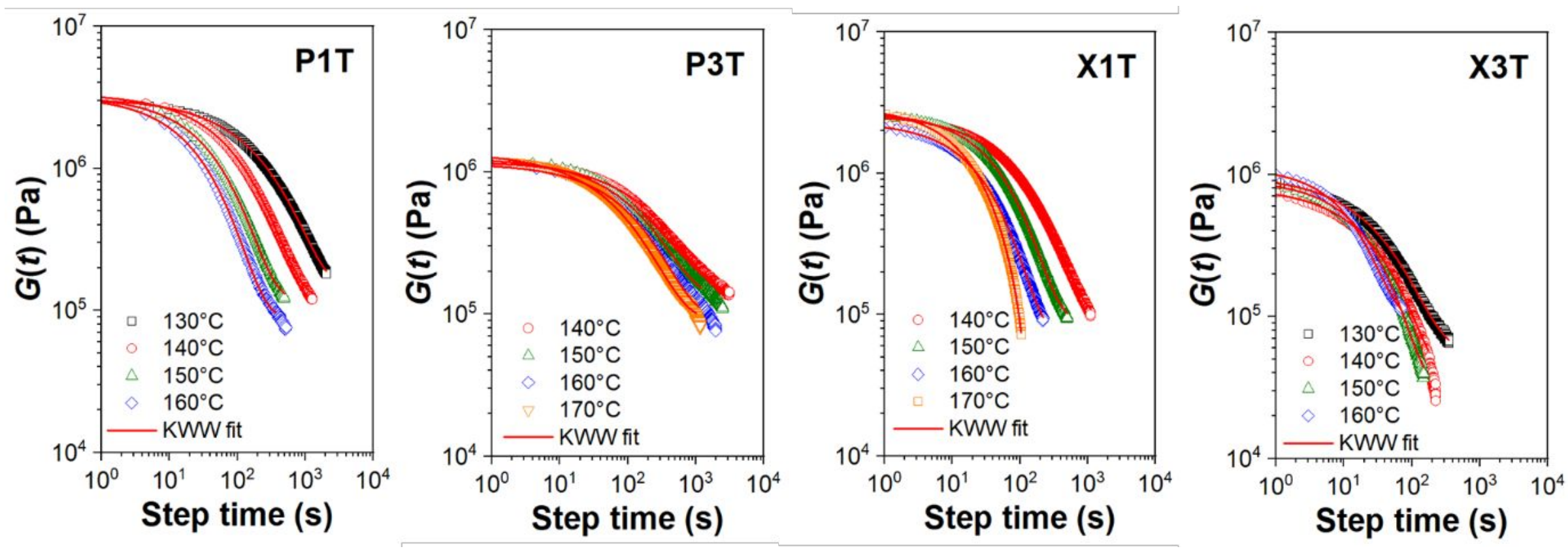

Figure S17. Stress relaxation curves of CANs at different temperatures and the results of fitting according to the Kohlrausch-William-Watts (KWW) stretched exponential decay.

Table S2. Results of stress relaxation curves fitting according to the Kohlrausch-William-Watts (KWW) stretched exponential decay

**Sample ID**	**Temperature (°C)**	**Kohlrausch-Williams-Watts**			
		***τ** *** **(s)**	***β***	**<*τ*> (s)**	**R^2**
P1T	130	190	0.60	286	0.999
	140	83	0.63	118	0.999
	150	41	0.68	53	0.999
	160	25	0.69	32	0.999
P3T	140	199	0.61	293	0.997
	150	144	0.60	217	0.992
	160	118	0.63	167	0.992
	170	93	0.63	132	0.993
X1T	140	86	0.62	124	0.999
	150	47	0.70	59	0.999
	160	28	0.72	35	0.999
	170	21	0.71	26	0.999
X3T	130	29	0.60	44	0.999
	140	26	0.64	36	0.998
	150	21	0.73	26	0.999
	160	12	0.80	14	0.999

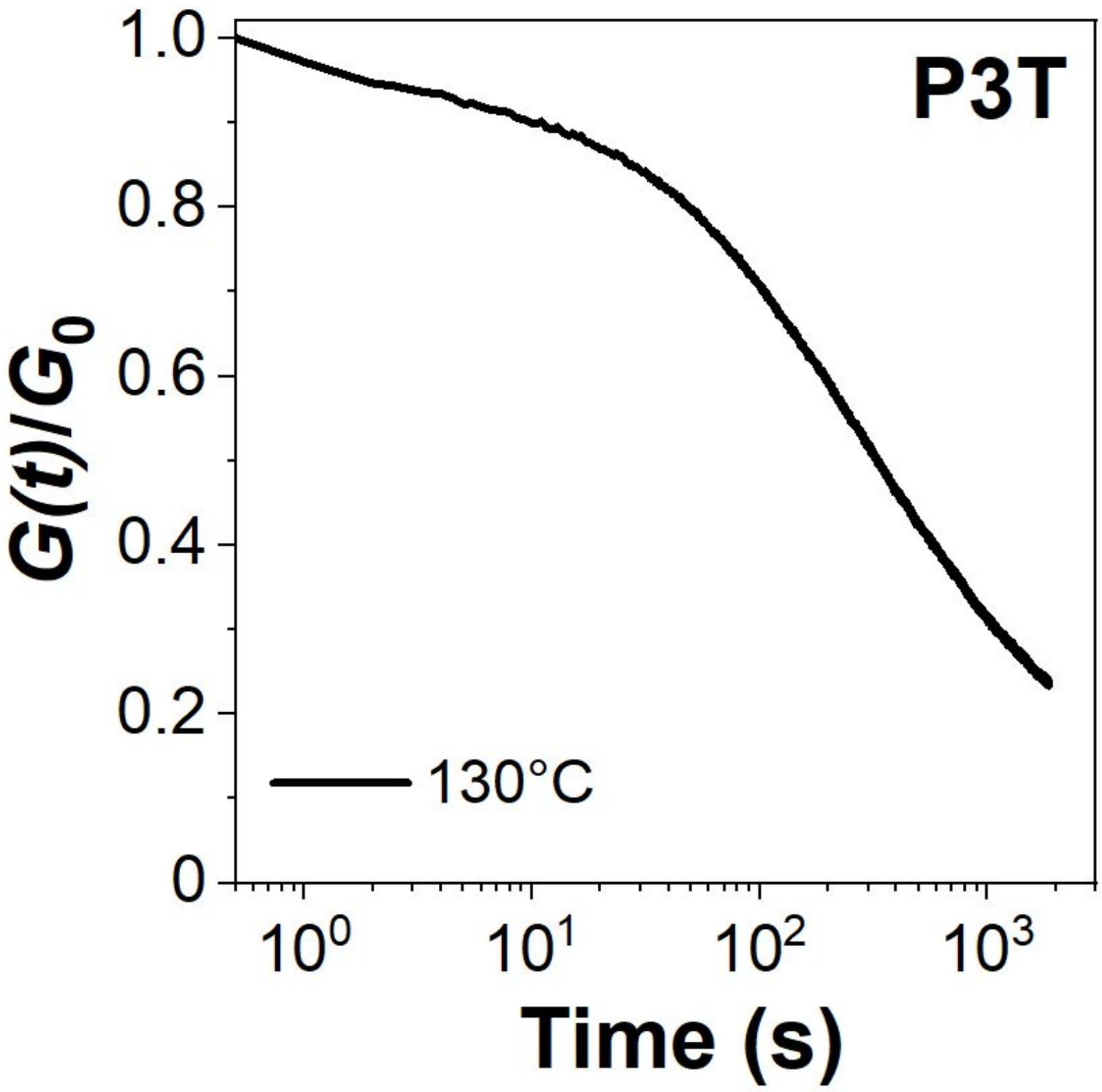

Figure S18 Normalized stress relaxation curve of P3T at 130 °C.

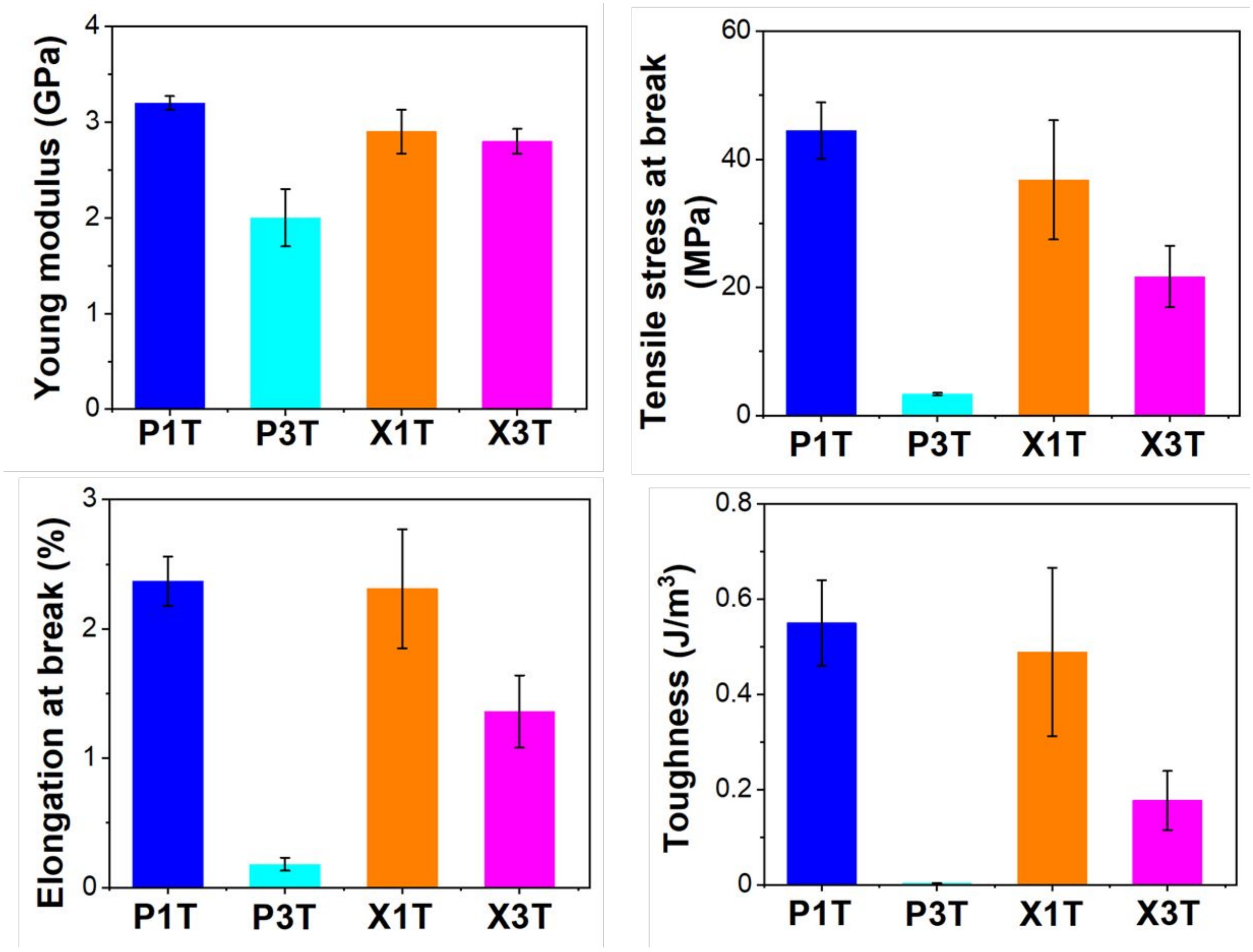

Figure S19. Young modulus, tensile stress at break, elongation at break and toughness of prepared CANs. Error bars represent standard deviation (n>6). The toughness for P3T sample was $5\cdot10^{-5}$ J/m^3, as data shows this sample was extremely brittle.

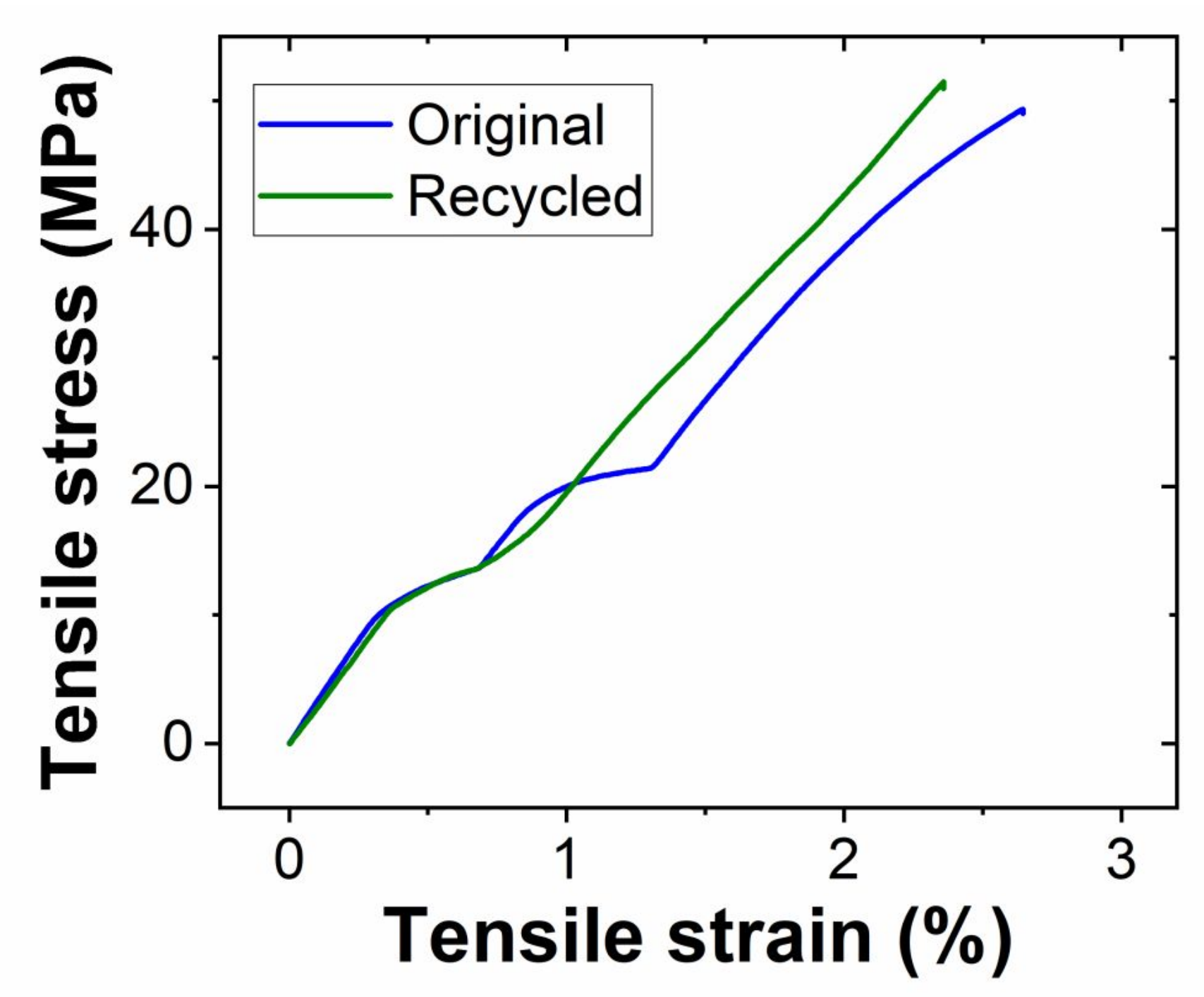

Figure S20. Tensile test: stress-strain curves of P1T before (original) and after (recycled) reprocessing.

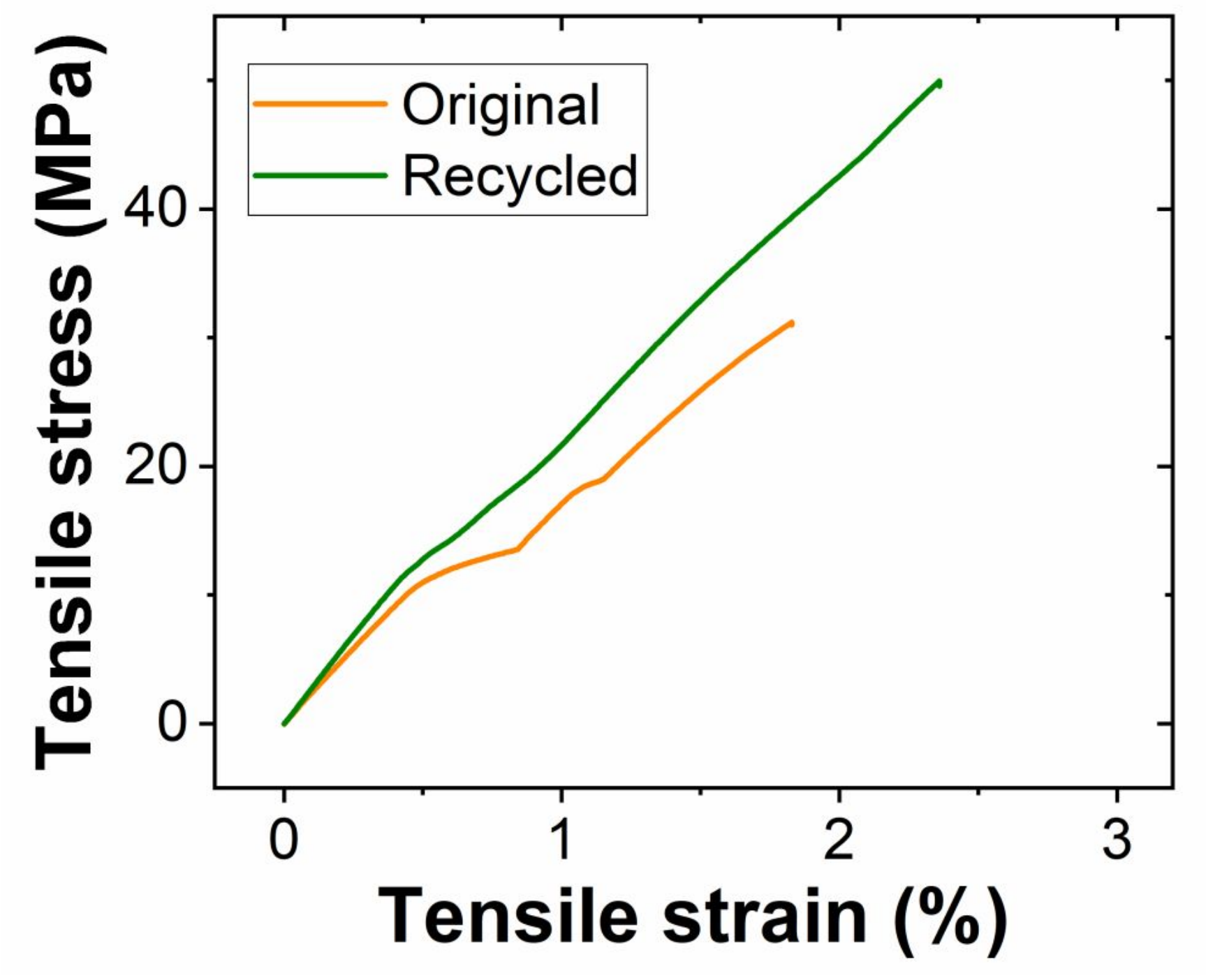

Figure S21. Tensile test: stress-strain curves of X1T before (original) and after (recycled) reprocessing.
